# ﻿An updated, illustrated inventory of the marine fishes of the US Virgin Islands

**DOI:** 10.3897/zookeys.1103.83795

**Published:** 2022-06-01

**Authors:** D. Ross Robertson, Carlos J. Estapé, Allison M. Estapé, Lee Richter, Ernesto Peña, Benjamin Victor

**Affiliations:** 1 Smithsonian Tropical Research Institute, Balboa, Panama Smithsonian Tropical Research Institute Balboa Panama; 2 197 Gulfview Drive, Islamorada, Florida, 33036, USA Unaffiliated Islamorada United States of America; 3 National Park Service, 1300 Cruz Bay Creek, St. John, VI 00830, Virgin Islands, USA National Park Service St. John Virgin Islands (USA); 4 Ocean Science Foundation, 4051 Glenwood, Irvine, CA 92604, USA Ocean Science Foundation Irvine United States of America; 5 Guy Harvey Research Institute, Nova Southeastern University, 8000 North Ocean Drive, Dania Beach, FL 33004, USA Nova Southeastern University Dania Beach United States of America

**Keywords:** Biodiversity, checklist, citizen science, DNA-barcode, photographic voucher, SCUBA survey

## Abstract

The US Virgin Islands (USVI) include St. John and St. Thomas on the Puerto Rican Platform (PRP) and St. Croix, isolated by 2000 m deep water 45 km south of that platform. Previous inventories of the marine fishes of these islands include a comprehensive 2014 checklist of the fishes of St. Croix and a list of the fishes of the PRP produced in 2000. The latter list noted the locations of many records of the plateau’s fishes, allowing the construction of a combined inventory for St. John and St. Thomas. Those two islands are treated here as a single faunal unit because they are only 3.5 km apart on a shared shallow shelf with various islets and reefs in between. Here we provide updated information on those two USVI (St. Croix and St. John-Thomas) marine fish faunas. The additions to the St. Croix and St. John-Thomas inventories presented here are based on a combination of information from the two sources indicated above, more recent publications dealing with those faunas, a review of location records on various online sources of biogeographic data, and voucher photographs taken of fishes in the field by authors of this paper and other citizen scientists. This assessment increased the known fauna of St. Croix by 7.5% to 585 species. The inventory for St. John-Thomas increased by 39.9% from 401 species on the 2000 PRP list to 561 with the inclusion of records from other sources. On-site mtDNA (COI) barcodes are available for approximately one-third of the species of the St. John-Thomas fauna, but for only one species collected at St. Croix. A set of underwater photographs of 372 species (34 of them representing the sole record of a species) from St. John-Thomas and of 11 shallow-water species added to the St. Croix fauna is included. These represent occurrence vouchers and also are intended to facilitate future work that builds on the present compendium.

## ﻿Introduction

The United States Virgin Islands (USVI) comprise a US territory adjacent to Puerto Rico, in the northeast Caribbean, that includes three large, inhabited islands, St. John, St. Thomas and St. Croix, and approximately 50 smaller islands and cays around them. The former two are situated only 3.5 km apart, in the center of the Puerto Rico Plateau (PRP), which has an area approximately twice the 9,100 km^2^ of Puerto Rico Island and extends ~ 150 km eastwards from Puerto Rico. St. Croix is located south of St. John and St. Thomas, on its own insular platform, which is separated by 45 km of deep water from the southern edge of the PRP.

The fish fauna of St. Croix was comprehensively reviewed by Smith-Vaniz and Jelks (2014), who built upon an older list by Clavijo et al. (1980), using their own extensive collections of shallow fishes of the Buck Island Reef National Monument on the northern side of St. Croix (Smith-Vaniz et al. 2006), and a review of literature and examination of specimens of fishes collected at St. Croix that are lodged in various museums. In 2000, George Dennis produced an extensive (244 page; 500+ sources cited) U.S. Geological Survey report based on collections and observational records for marine and brackish-water fish from Puerto Rico, St. John and St. Thomas, and other islands on the PRP. Although never formally published in a scientific journal, and no longer available through the USGS source cited by Dennis et al. (2004), that compendium is available online (Dennis 2000).

Here we add new information to update the 2014 list for St. Croix and assemble an inventory for St. John and St. Thomas that includes and expands on data for those two islands contained in Dennis (2000). We extracted the additional information from museum records in online sources of biogeographic data, publications produced since Dennis (2000), digital images of live fishes obtained at the USVI, plus our recent collections and mtDNA barcode records obtained from the database BOLD. The great majority of the species in this compendium are marine, plus we include a small number of species found in fresh to brackish waters.

## ﻿Materials and methods

### ﻿Study sites

St. Croix is a 215 km^2^ island in the northeast corner of the Caribbean. It is isolated by ~ 45 km of deep water from the Puerto Rican Platform (**PRP**). Other islands of the Lesser Antilles chain lie within ~ 150 km to the east and southeast of St. Croix. The surrounding shallow (above ~ 150 m depth) shelf of St. Croix, extending almost 20 km eastward, has approximately the same area as the island. In addition to exposed and sheltered coral reefs and soft bottoms, the island has extensive areas of seagrasses and mangroves.

St. John (area 50 km^2^) and St. Thomas (area 83 km^2^) are situated in the center of the shallow (to ~ 150 m deep) tongue of the PRP that extends 150 km eastwards from Puerto Rico. St. Thomas is closest to and 64 km from the main island of Puerto Rico. St. John and St. Thomas are separated from each other by only 3.5 km of water shallower than 20 m deep, with scattered islets and shallow reefs in between them. They have a similar range of habitats as St. Croix, with large areas of both sheltered and deeper shelf-edge coral reefs, rocky shores, seagrass beds and mangroves. Due to their proximity and similarity of habitats we treat them here as a single unit (hereafter St. John-Thomas). The shallow PRP associated with St. John-Thomas extends ~ 25 km north and ~ 15 km south of those islands and covers an area of ~ 2,100 km^2^ (Rohmann et al. 2005).

Suppl. material 2: File S1 shows the bathymetry of bottom habitats on the above-150 m shelves of the USVI. The shelf area of the St. John-Thomas EEZ is not only much larger than that of St. Croix but also differs from the latter in containing a much greater diversity of areas of different depths. There are large expanses, in both absolute and relative terms, of habitat between 40–60 m deep to the north of St. Thomas and to the south of both islands. In contrast, most of the smaller shelf of St. Croix is shallower than 20 m deep.

### ﻿Data sources

We reviewed and cited only publications from which we extracted information relating to the USVI fishes that were published after those cited by Dennis (2000) for St. John-Thomas, and after that by Smith-Vaniz and Jelks (2014) for St. Croix, plus a few earlier publications that contained additional relevant information.

Smith-Vaniz and Jelks (2014) published a comprehensive, annotated checklist of 544 fishes known from St. Croix. That checklist was based, in large part, on the yield of fishes from 106 rotenone stations obtained by Smith-Vaniz et al. (2006) and by later workers to document the shallow cryptobenthic fauna. That 2014 list identified questionable records, a few of which, as we show, have turned out to be valid. Smith-Vaniz and Jelks (2014). That checklist also excluded deep-water fishes not found above 200 m as well as Exocoetids and Myctophids. For completeness we have included any such species recorded by other sources among the additions noted here. We used the 2014 list of valid species and reviewed fishes listed by other surveys: a SCUBA study of the shallower parts (30–50 m depth) of a mesophotic coral ecosystem at the eastern end of the shelf (García-Sais et al. 2014); two JSL submersible dives off St. Croix to 30–600 m (Nelson and Appeldoorn (1985); and two ROV dives off St. Croix at depths greater than 800 m (Quattrini et al. 2017). In addition, we reviewed the records of fish species from St. Croix available from various online sources: the aggregators GBIF (https://www.gbif.org/), FishNet2 (http://www.fishnet2.net/), iDigBio (https://www.idigbio.org/portal), OBIS (https://obis.org/) and Vertnet (http://vertnet.org/), and the American Museum of Natural History (AMNH; https://www.amnh.org/research/vertebrate-zoology/ichthyology). Those searches were made within a quadrat with latitudinal limits of 17.62°N to 17.85°N, and longitudinal limits of -64.4°W to -65.0°W, encompassing St. Croix and all of its platform. The sources of St. Croix records produced by those online searches were evaluated and museum records within the known geographic range of various species were accepted. Evaluation of individual records is necessary because aggregator information includes significant numbers of erroneous records.

**Figure 1.**
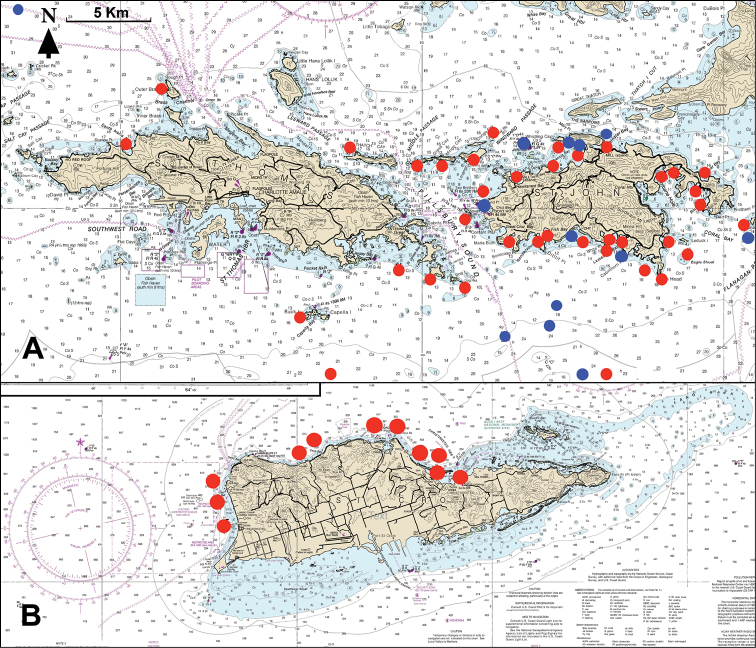
**A** dive sites generating fish-occurrence data at St. John and St. Thomas islands. Dive sites of CJE and AME are indicated by red symbols, and of other sources of voucher photographs by blue symbols. Note that some close-proximity sites are indicated by a single symbol. Symbols at the northern and southern edges of Fig. 1A are representative only, as their latitudes are outside the area of the map **B** dive sites of CJE and AME generating data at St. Croix. See Suppl. material 3: File S2A, B and Suppl. material 4: File S3 for further information. Base map in both cases: NOAA Chart 25641.

Finally, the list includes shallow-reef fishes photographed by authors AME and CJE during a month spent at the island from 19 December 2020 to 13 January 2021. Suppl. material 3: File S2A presents a list, with georeferenced locations, of the 11 dive sites at which they together made 25 dives (total 47 hours duration per person) during that period (see also Fig. 1B and Suppl. material 4: File S3, a Google Earth © KMZ file that shows, for each of those sites, its location and georeferenced coordinates, and the number of dives and total dive time spent at that site). These photographs document a few species not previously recorded at the island, plus several not accepted by Smith-Vaniz and Jelks (2014) due to a lack of reliable information.

For St. John-Thomas we extracted a list of 401 species listed at those islands by Dennis (2000) and reviewed various publications dealing with fish records at and near those islands that were subsequently produced. Finally, we also used the same online data sources as for St. Croix (see above) to obtain records of fishes from the part of the Exclusive Economic Zone of the USVI that includes St. John-Thomas and extends between the northern and southern edges of the PRP. That irregularly shaped EEZ was obtained from Marineregions.org, which provides a standard set of global maps of EEZs (https://www.marineregions.org/eezsearch.php).

CJE and AME spent six months between 3 November 2020 and 29 May 2021 diving at both islands and photographing fishes to obtain voucher images of as many members of those islands’ marine fish fauna as possible. File S2A presents a list, with georeferenced locations, of their dive sites at St. John (37) and St. Thomas (12), at which they made 113 joint dives (involving multiple dives at some sites) totaling 221 hours per person and 37 dives totaling 37 hours per person, respectively. Fig. 1A is a map with those 49 dive sites at St. John-Thomas indicated and File S2 provides additional information. Fig. 1A (and see File S2B) also indicates the location of sites from other sources at which additional species not recorded by CJE and AME were documented photographically by other divers.

### ﻿Reef-associated bony fishes of the USVI

Greater Caribbean (GC) reef systems have reef-fish faunas that are dominated by members of typical, shallow-reef families of bony fishes extending down to depths of ~ 250–300 m (Baldwin et al. 2018). Here we focus on species belonging to those families, which have traditionally been viewed as reef fishes. We classed species living entirely or largely below 40 m depth as belonging to the deep-reef subset. Species classed here as shallow include both species restricted to depths shallower than 40 m and those with depth ranges that extend above and below that level. These reef-associated fishes include not only benthic and demersal species found on hard-reef substrata, but also pelagic fishes that facultatively associate with reefs and benthic and demersal species that live on soft bottoms within and immediately around the fringes of reefs. Benthic species (e.g., eels, flatfishes) are restricted to life on and in different types of substrata, while demersal species (e.g., snappers and grunts) use both substratum habitats and the water column. Cryptobenthic species are visually cryptic and typically small. We followed Brandl et al. (2018) in classifying families dominated by small cryptobenthic coral-reef species as Core Coral Reef Fish families (CCRFs).

We also evaluate the ecological and zoogeographic composition of the two USVI fish faunas (St. Croix and St. John-Thomas) compared to the complete checklist of the regional fauna of reef-associated bony fishes, which includes 992 species in 342 genera and 84 families (Robertson and Tornabene 2021). These aspects of the fauna of the USVI are also compared with results from another recent comprehensive survey of the fish fauna of nearby Sint Eustatius, which is 170 km from St. Croix (Robertson et al. 2020).

### ﻿mtDNA-barcode coverage of fishes collected in the USVI and Puerto Rico

Relatively few small marine locations have been comprehensively sampled for fish DNA barcoding, i.e., tissues sequenced for the mtDNA COI marker as a standard for identifying fishes, as compiled in the Barcode of Life Database, BOLD (Ward et al. 2009). Notably, BOLD not only includes a wide variety of projects, most of which are publicly available, but also regularly harvests all available COI sequences from GenBank. In contrast, GenBank does not harvest from BOLD, and BOLD sequences are generally submitted to GenBank only by request. As a result, only a fraction (~ 15% for GC fishes) of COI sequences on BOLD also are present on GenBank, despite its widespread use as the sole source for barcoding studies. BOLD further differs from GenBank by applying quality control to sequences and taxon identifications as data is entered, including sequences harvested from GenBank. It also has post-hoc quality control via a tagging and comment option on individual records. BOLD also includes a large number of private sequences, which can be assessed to a limited degree (with some metadata removed) via the BIN portal, which compiles all records, public and private, within a lineage, assigns a code, and presents some statistics, especially variance and nearest neighbor distances, as well as countries of origin.

The BOLD BIN code is a key advance enabling the compilation and comparison of mtDNA barcoding lists, since it supplies an independent identifier for a monophyletic genetic lineage, which is not the same as a species name. BOLD creates **BINs** (Barcode Index Numbers) by clustering barcode sequences algorithmically. The BIN often represents a particular species, but there are many exceptions to the “one-species, one BIN” concept: either multiple BINs per species, indicating genetically divergent populations within species (usually allopatric, but not always), a subset of which are putative new cryptic species awaiting morphological confirmation; or shared BINs by two or more species that retain shared or closely related haplotypes due to a short time since speciation, to incomplete lineage sorting, or to a small degree of hybridization.

Our broad assessment suggests that BOLD has a BIN that can be assigned (with widely varying degrees of confidence) to ~ 900 species of shallow-dwelling, reef-associated bony fishes from the GC. A list of sequences obtained in a particular area is obtained from BOLD by using a vector map in its search engine. The resulting list is from public projects (including all GenBank COI sequences), as well as whichever private projects the user has permission to access (often granted by an email request to the source of the sequence). In our case, we have been given access to all of the larger private projects in the region and barcodes for the vast majority (~ 90%) of sequence records in BOLD that could be evaluated in their respective BINs. The list of records from the geographic-area search on BOLD are individual sequences with metadata (including GenBank number if a sequence has one) and photographs of specimens (when available), together with a link to the BIN code to which it belongs. The species name originally submitted for each is preserved, and the accuracy of the assignment can be assessed by examining the BIN to which it belongs, which has details on the various names applied to sequences in the BIN and by whom and where they were obtained. Accuracy assessments are critical, especially for more obscure species, since a “majority rules” decision is often inaccurate due to multiple identifications by inexperienced contributors, the tendency to repeat the species-level identification made by others as a shortcut, and the practice of assigning species-level names to submitted records that are from eggs, larvae, isolated tissue, or fish-market specimens. GenBank records are harvested by BOLD with whatever name is assigned in GenBank, often a preliminary one from submission, rather than the one later corrected or published in the subsequent literature.

## ﻿Results

### ﻿The island faunas

St. Croix: The checklist of Smith-Vaniz and Jelks (2014) included 544 species from 280 genera in 94 families. We obtained records of 41 species (belonging to 39 genera and 35 families; see Table 1) that were not included on that checklist, an increase of 7.5% in the number of species. Those new records included 19 deep-living species, six of them (11.1% of all deep species at St. Croix) resulting solely from observations by the JSL submersible (Nelson and Appeldoorn 1985; García-Sais et al. 2014) and an ROV (Remotely Operated Vehicle; Quattrini et al. 2017). It should be noted that almost all of that group belong to very deep taxa specifically excluded by Smith-Vaniz and Jelks (2014) from their list, which was focused primarily on shallower fishes. The remaining 22 species are shallow-water, reef-associated fishes. Ten of the latter group were photographed by AME and CJE (Table 1; Suppl. material 1: Plate S1). These additions include three species (*Eucinostomusmelanopterus*, *Coryphopterusglaucofrenum* and *Opistognathusmacrognathus*) that Smith-Vaniz and Jelks (2014) referred to but did not include in their checklist due to lack of confirmed records. Records of two mobulid rays consisted of identified photographs/videos provided by Mantatrust.org (https://www.mantatrust.org/) that were inspected by DRR. The list (Table 1, which includes source information) also includes records from museum collections that provide online data directly or indirectly through aggregators, which were included if consistent with the known geographic range of each of those species.

**Table 1.** Species of fishes added to the St. Croix checklist of fishes of Smith-Vaniz and Jelks (2014).

Scientific name	Common name	Deep	Image plate	Literature source	Online source
**Antennariidae**					
*Fowlerichthysocellatus* (Bloch & Schneider, 1801)	Ocellated Frogfish				TNHCi
**Bathygadidae**					
*Gadomusarcuatus* (Goode & Bean, 1886)	Doublethread Grenadier	yes		6	
**Blenniidae**					
*Hypleurochiluspseudoaequipinnis* Bath, 1994	Oyster Blenny		S1		
**Bramidae**					
*Eumegistusbrevorti* (Poey, 1860)	Tropical Pomfret	yes			FlMNH
**Chaenopsidae**					
*Emblemariopsisleptocirris* Stephens, 1970	Fine-cirrus Blenny		S1		
**Chimaeridae**					
*Chimaeracubana* Howell Rivero, 1936	Cuban Chimaera	yes		1	
**Etmopteridae**					
*Etmopterushillianus* (Poey, 1861)	Caribbean Lantern Shark	yes			FlMNH
**Exocoetidae**					
*Cheilopogonmelanurus* (Valenciennes, 1847)	Atlantic Flyingfish				CF
*Cypseluruscomatus* (Mitchill, 1815)	Clearwing Flyingfish				CF
**Gempylidae**					
*Lepidocybiumflavobrunneum* (Smith, 1843)	Escolar	yes			NOAA
*Nesiarchusnasutus* Johnson, 1862	Black Gemfish	yes			NMNH
**Gerreidae**					
*Eucinostomusmelanopterus* (Bleeker, 1863)	Flagfin Mojarra		S1	5,7*	
**Gobiesocidae**					
*Acyrtuslanthanum* Conway, Baldwin & White, 2014	Orange-spotted Clingfish				FlMNH
**Gobiidae**					
*Coryphopterusglaucofraenum* Gill, 1863	Bridled Goby		S1	2,5.7*	
*Coryphopteruskuna* Victor, 2007	Kuna Goby		S1		
*Oxyurichthysstigmalophius* (Mead & Böhlke, 1958)	Spotfin Goby		S1		NOAA
**Kyphosidae**					
*Kyphosuscinerascens* (Forsskål, 1775)	Topsail Seachub		S1		
**Macrouridae**					
*Nezumiaaequalis* (Günther, 1878)	Atlantic Blacktip Grenadier	yes		6	
**Malakichthyidae**					
*Veriluspseudomicrolepis* (Schultz, 1940)	False-smallscale Bass	yes			CAS
**Mobulidae**					
*Mobulacfbirostris*	Giant Manta			4	
*Mobulatarapacana* (Philippi, 1892)	Sicklefin Devil Ray			4	
**Muraenidae**					
*Gymnothoraxnigromarginatus* (Girard, 1858)	Blackedge Moray				CAS
**Nemichthyidae**					
*Nemichthyscurvirostris* (Strömman, 1896)	Spottedbelly Snipe Eel	yes		6	
**Neoscopelidae**					
*Neoscopelusmicrochir* Matsubara, 1943	Shortfin Blackchin	yes		6	
**Ophichthidae**					
*Myrophispunctatus* Lütken, 1852	Speckled Worm Eel				MCZ
**Ophidiidae**					
*Monomitopusagassizii* (Goode & Bean, 1896)	Threespine Cusk-eel	yes			MCZ
**Opistognathidae**					
*Opistognathusmacrognathus* Poey, 1860	Banded Jawfish		S1	5,7*	
**Paralichthyidae**					
*Syaciummicrurum* Ranzani, 1842	Channel Flounder		S1		
**Peristediidae**					
*Peristedionlongispatha* Goode & Bean, 1886	Widehead Armored Searobin	yes			MCZ
**Pleuronectidae**					
*Poecilopsettainermis* (Breder, 1927)	Unarmed Deepwater Dab	yes			CAS, NMNH
**Polymixiidae**					
*Polymixianobilis* Lowe, 1836	Noble Beardfish	yes		3	
**Scombropidae**					
*Scombropsoculatus* (Poey, 1860)	Atlantic Scombrops	yes			FlMNH
**Sparidae**					
*Calamuscalamus* (Valenciennes, 1830)	Saucereye Porgy			5	
**Squalidae**					
*Cirrhigaleusasper* (Merrett, 1973)	Roughskin Spiny Dogfish	yes			FlMNH
**Stomiidae**					
*Borostomiasmononema* (Regan & Trewavas, 1929)	Sickle Snaggletooth	yes		8	
**Synagropidae**					
*Synagropsbellus* (Goode & Bean, 1896)	Blackmouth Bass	yes		6	
**Syngnathidae**					
*Hippocampuserectus* Perry, 1810	Lined Seahorse				NCSM
**Synodontidae**					
*Synodusfoetens* (Linnaeus, 1766)	Inshore Lizardfish				ANSP
*Trachinocephalusmyops* (Forster, 1801)	Snakefish		S1		
**Trachipteridae**					
*Zucristatus* (Bonelli, 1820)	Scalloped Ribbonfish	yes		8	
**Tripterygiidae**					
*Enneanectesquadra* Victor, 2017	Squaretail Triplefin				FlMNH

**Notes**: Deep – restricted to depths below 40 m. Image Plate – see Suppl. material 1: Plate S1 for voucher images. Literature source – 1 Bunckley-Williams and Williams (2004); 2 Garcia-Sais et al. (2014); 3 Nelson and Appeldoorn (1985); 4 Mantatrust.org; 5 Pittman et al. (2008); 6 Quatrinni et al. (2017); 7 Smith-Vaniz and Jelks (2014) (asterisk indicates a species that was discussed by not included by those authors); 8 Clavijo et al. (1980). Online source - TNHCi (University of Texas at Austin, Biodiversity Center, Ichthyology collection; FlMNH (Florida Museum of Natural History); CF (Biological observations from the Dana Expedition Reports); NOAA (National Oceanographic and Atmospheric Administration); CAS (California Academy of Sciences); MCZ (Museum of Comparative Zoology); NMNH (National Museum of Natural History); NCSM (North Carolina State Museum of Natural Sciences); ANSP (Academy of Natural Sciences of Philadelphia). *Coryphopterus*: Smith-Vaniz et al. (2014) concluded that *C.tortugae*, but not *C.glaucofrenum*, was present at St. Croix. However, CJE and AME photographed both species at St. Croix, illustrated in Suppl. material 1: Plate S1.

St. John-Thomas: Table 2 presents a list of species recorded from those islands together with the source(s) of each record (images, publications, DNA barcodes, or online museum records) and which species have a voucher image in the supplementary plates (Suppl. material 1: Plates S2–S18). In addition, for uncommon species (those encountered by AME, CJE, LR, or third-party photographers at three or fewer dive sites) the names of the sites at which those uncommon species were found are included, to aid future investigations. Dennis (2000) also included information on species that were collected using the ichthyocide Rotenone (see Table 2). Smith-Vaniz and Jelks (2014) list for St. Croix also included some species recorded at these St. John-Thomas as a result of collections using that ichthyocide. Two ROV dives of Quattrini et al. (2017) and four dives (including one to only 50 m depth on the PRP a little to the north of St. Thomas) by the JSL submersible at St. John-Thomas (Nelson and Appeldoorn 1985; Garcia-Sais 2005) yielded 75 species records. Of those 19 were of deep-living species, with 14 (28%) representing sole-source records of the 50 deep-living fishes currently known to occur at St. John-Thomas.

**Table 2.** Checklist of the fishes of St. John-Thomas islands.

Scientific name	Common name	Image Plate	Literature source	Online source	Uncommon (site code)	Ichthyocide	DNA
**Acanthuridae**							
*Acanthuruschirurgus* (Bloch, 1787)	Doctorfish	S2	2,4,8	1		1	
*Acanthuruscoeruleus* Bloch & Schneider, 1801	Blue Tang	S2	2,4,5,8	1		1	YES
*Acanthurustractus* Poey, 1860	Northern Ocean Surgeonfish	S2	2,4,5,8	1		1	YES
**Achiridae**							
*Gymnachirusnudus* Kaup, 1858	Flabby Sole	S2	2,11	1	SJ5, SJ18, SJ25		YES
**Aetobatidae**							
*Aetobatusnarinari* (Euphrasen, 1790)	Spotted Eagle Ray	S2	2	1			
**Albulidae**							
*Albulagoreensis* Valenciennes, 1847	Senegalese Bonefish			NOAA-BOLD			YES
*Albulavulpes* (Linnaeus, 1758)	Bonefish		2,4	1			YES
**Anguillidae**							
*Anguillarostrata* (Lesueur, 1817)	American Eel		6	1			
**Antennariidae**							
*Antennariusmultiocellatus* (Valenciennes, 1837)	Longlure Frogfish	S2	2	1		1	
*Antennariuspauciradiatus* Schultz, 1957	Dwarf Frogfish		2	1			
*Histriohistrio* (Linnaeus, 1758)	Sargassumfish	S2	12		O22		
**Apogonidae**							
*Apogonaurolineatus* (Mowbray, 1927)	Bridle Cardinalfish	S2	2,4	1			YES
*Apogonbinotatus* (Poey, 1867)	Barred Cardinalfish	S2	2,4	1		1	
*Apogonlachneri* Böhlke, 1959	Whitestar Cardinalfish	S2	2,4	1	SJ2	1	
*Apogonmaculatus* (Poey, 1860)	Flamefish	S2	2,4	1		1	YES
*Apogonphenax* Böhlke & Randall, 1968	Mimic Cardinalfish	S2	2,11			1	
*Apogonplanifrons* Longley & Hildebrand, 1940	Pale Cardinalfish	S2	2	1		1	
*Apogonpseudomaculatus* Longley, 1932	Twospot Cardinalfish		2,4	1			
*Apogonquadrisquamatus* Longley, 1934	Sawcheek Cardinalfish	S2	2,4	1	SJ22, SJ25	1	YES
*Apogonrobinsi* Böhlke & Randall, 1968	Roughlip Cardinalfish		2			-1	
*Apogontownsendi* (Breder, 1927)	Belted Cardinalfish	S2	2,4	1		1	YES
*Astrapogonpuncticulatus* (Poey, 1867)	Blackfin Cardinalfish	S2	2	1			YES
*Astrapogonstellatus* (Cope, 1867)	Conchfish	S2	2,4	1	SJ5, SJ13		YES
*Paroncheilusaffinis* (Poey, 1875)	Bigtooth Cardinalfish		2	1			
*Phaeoptyxconklini* (Silvester, 1915)	Freckled Cardinalfish	S2	2	1		1	YES
*Phaeoptyxpigmentaria* (Poey, 1860)	Dusky Cardinalfish	S2	2	1		1	YES
*Phaeoptyxxenus* (Böhlke & Randall, 1968)	Sponge Cardinalfish	S2	2	1		1	YES
*Zapogonevermanni* (Jordan & Snyder, 1904)	Oddscale Cardinalfish	S2			SJ22		YES
**Atherinidae**							
*Atherinaharringtonensis* Goode, 1877	Reef Silverside		2	1		1	YES
*Atherinomorusstipes* (Müller & Troschel, 1848)	Hardhead Silverside	S2	2,6	1		1	
**Aulostomidae**							
*Aulostomusmaculatus* Valenciennes, 1841	Atlantic Trumpetfish	S2	2,4	1		1	
**Balistidae**							
*Balistescapriscus* Gmelin, 1789	Gray Triggerfish	S3	2				
*Balistesvetula* Linnaeus, 1758	Queen Triggerfish	S3	2,4,5,8	1		1	YES
*Canthidermissufflamen* (Mitchill, 1815)	Ocean Triggerfish	S3	2	1	SJ33		
*Melichthysniger* (Bloch, 1786)	Black Durgon	S3	2,4	1	SJ33		
*Xanthichthysringens* (Linnaeus, 1758)	Sargassum Triggerfish	S3	2,5	1	SJ33		
**Belonidae**							
*Ablenneshians* (Valenciennes, 1846)	Barred Needlefish	S3					
*Platybeloneargalusargalus* (Lesueur, 1821)	Keeltail Needlefish	S3	2	1		1	
*Strongyluratimucu* (Walbaum, 1792)	Timucú		2,6	1			
*Tylosurusacus* (Lacepède, 1803)	Atlantic Agujón			FlMNH, MCZ			
*Tylosuruscrocodilus* (Péron & Lesueur, 1821)	Houndfish	S3	2	1			
**Blenniidae**							
*Entomacrodusnigricans* Gill, 1859	Pearl Blenny	S3	2	1		1	YES
*Hypleurochiluspseudoaequipinnis* Bath, 1994	Oyster Blenny	S3	2,11	1			YES
*Hypleurochilusspringeri* Randall, 1966	Orangespotted Blenny	S3	2	1			
*Hypsoblenniusinvemar* Smith-Vaniz & Acero P., 1980	Tessellated Blenny	S3	11	1	ST11		YES
*Ophioblenniusmacclurei* (Silvester, 1915)	Redlip Blenny	S3	2,4	1		1	YES
*Parablenniusmarmoreus* (Poey, 1876)	Seaweed Blenny	S3	2,4	1		1	YES
*Scartellacristata* (Linnaeus, 1758)	Molly Miller	S3	2,4	1		1	YES
**Bothidae**							
*Bothuslunatus* (Linnaeus, 1758)	Peacock Flounder	S3	2,4	1		1	
*Bothusmaculiferus* (Poey, 1860)	Mottled Flounder	S3			SJ3, SJ5, SJ28		
*Bothusocellatus* (Agassiz, 1831)	Eyed Flounder	S3	2,4	1			
*Bothusrobinsi* Topp & Hoff, 1972	Twospot Flounder		2,3				
**Bythitidae**							
*Calamopteryxgoslinei* Böhlke & Cohen, 1966	Longarm Brotula		2			-1	
*Grammonusclaudei* (de la Torre y Huerta, 1930)	Reef-cave Brotula		2	1		-1	
*Petrotyxsanguineus* (Meek & Hildebrand, 1928)	Redfin Brotula		2	1		-1	
**Callionymidae**							
*Callionymusbairdi* Jordan, 1888	Lancer Dragonet	S3	2,4	1			YES
*Chalinopspauciradiatus* (Gill, 1865)	Spotted Dragonet	S3	2	1	SJ28, SJ3, SJ5		YES
**Carangidae**							
*Alectisciliaris* (Bloch, 1787)	African Pompano	S4	2	1	ST1, SJ13		
*Caranxbartholomaei* Cuvier, 1833	Yellow Jack	S4	2,4	1			
*Caranxcrysos* (Mitchill, 1815)	Blue Runner	S4	2,4	1			
*Caranxhippos* (Linnaeus, 1766)	Crevalle Jack	S4			SJ29		
*Caranxlatus* Agassiz, 1831	Horse-eye Jack	S4	2,6	1			
*Caranxlugubris* Poey, 1860	Black Jack	S4	2,4,5,8	1	SJ33		
*Caranxruber* (Bloch, 1793)	Bar Jack	S4	2,4,8	1		1	
*Chloroscombruschrysurus* (Linnaeus, 1766)	Atlantic Bumper		2				
*Decapterusmacarellus* (Cuviers, 1833)	Mackerel Scad	S4	2	1			
*Decapteruspunctatus* (Cuvier, 1829)	Round Scad	S4	2	1			
*Decapterustabl* Berry, 1968	Redtail Scad	S4			SJ11		
*Elagatisbipinnulata* (Quoy & Gaimard, 1825)	Rainbow Runner	S4	2	1	SJ33		
*Oligoplitessaurussaurus* (Bloch & Schneider, 1801)	Leatherjack		2	1			
*Selarcrumenophthalmus* (Bloch, 1793)	Bigeye Scad	S4	2	1	SJ13		
*Selenebrownii* (Cuvier, 1816)	Caribbean Moonfish		2	1			
*Selenevomer* (Linnaeus, 1758)	Lookdown			FlMNH			
*Serioladumerili* (Risso, 1810)	Greater Amberjack		2,5				
*Seriolarivoliana* Valenciennes, 1833	Almaco Jack	S4	2	1	SJ16, SJ23		
*Trachinotusfalcatus* (Linnaeus, 1758)	Permit	S4	2	1	SJ22, SJ23		
*Trachinotusgoodei* Jordan & Evermann, 1896	Palometa	S4	2,4	1	SJ23, SJ15		
**Carcharhinidae**							
*Carcharhinusacronotus* (Poey, 1860)	Blacknose Shark	S4	1,2,10	1	SJ35, SJ27, ST7		
*Carcharhinusfalciformis* (Müller & Henle, 1839)	Silky Shark	S4			1, O1		
*Carcharhinusgalapagensis* (Snodgrass & Heller, 1905)	Galapagos Shark		2				
*Carcharhinuslimbatus* (Müller & Henle, 1839)	Blacktip Shark		1,2	1			
*Carcharhinuslongimanus* (Poey, 1861)	Oceanic Whitetip Shark			NMNH			
*Carcharhinusperezii* (Poey, 1876)	Reef Shark	S4	2,10	1	SJ13		
*Carcharhinusplumbeus* (Nardo, 1827)	Sandbar Shark			ANSP			
*Negaprionbrevirostris* (Poey, 1868)	Lemon Shark	S4	1,2,6,10	1	SJ12, O2		
*Rhizoprionodonporosus* (Poey, 1861)	Caribbean Sharpnose Shark		1,2,10	1			
**Centrophoridae**							
*Centrophorusuyato* (Rafinesque, 1810)	Little Gulper Shark			CAS			
**Centropomidae**							
*Centropomusensiferus* Poey, 1860	Swordspine Snook		6	1			
*Centropomusundecimalis* (Bloch, 1792)	Common Snook	S4	2,6	1			
**Chaenopsidae**							
*Acanthemblemariaaspera* (Longley, 1927)	Roughhead Blenny	S5	2	1	ST3		YES
*Acanthemblemariamaria* Böhlke, 1961	Secretary Blenny	S5	4	1			YES
*Acanthemblemariaspinosa* Metzelaar, 1919	Spinyhead Blenny	S5	2,4	1		1	YES
*Chaenopsislimbaughi* Robins & Randall, 1965	Yellowface Pikeblenny	S5	2,4	1			YES
*Chaenopsisocellata* Poey, 1865	Bluethroat Pikeblenny		2,4	1			
*Coralliozetuscardonae* Evermann & Marsh, 1899	Twinhorn Blenny	S5	11	1			YES
*Emblemariapandionis* Evermann & Marsh, 1900	Sailfin Blenny	S5	2,4	1			YES
*Emblemariavitta* Williams, 2002	Ribbon Blenny	S5	2,3	1	ST6	-1	YES
*Emblemariopsisbahamensis* Stephens, 1961	Blackhead Blenny	S5		1			YES
*Emblemariopsiscarib* Victor, 2010	Carib Blenny		2	1		-1	YES
*Emblemariopsisleptocirris* Stephens, 1970	Fine-cirrus Blenny	S5	2,11			-1	YES
*Emblemariopsisruetzleri* Tyler & Tyler, 1997	Ruetzler’s Blenny			BOLD, NMNH			YES
*Lucayablenniuszingaro* (Böhlke, 1957)	Arrow Blenny	S5			SJ18, SJ19		
**Chaetodontidae**							
*Chaetodoncapistratus* Linnaeus, 1758	Foureye Butterflyfish	S5	2,4,5,8	1		1	YES
*Chaetodonocellatus* Bloch, 1787	Spotfin Butterflyfish	S5	2,4	1			
*Chaetodonsedentarius* Poey, 1860	Reef Butterflyfish	S5	2,4,5,8	1			
*Chaetodonstriatus* Linnaeus, 1758	Banded Butterflyfish	S5	2,4	1		1	
*Prognathodesaculeatus* (Poey, 1860)	Longsnout Butterflyfish	S5	2,5,8	1			
*Prognathodesguyanensis* (Durand, 1960)	Guyana Butterflyfish		2,5,8,11				
**Chaunacidae**							
Chaunax pixtus Fowler, 1946	Uniform Gaper		5				
*Chaunaxsuttkusi* Caruso, 1989	Pale-cavity Gaper			CAS			
**Chlopsidae**							
*Chilorhinussuensonii* Lütken, 1852	Seagrass Eel		2	1			
*Kaupichthyshyoproroides* (Strömman, 1896)	False Moray		2	1		-1	
*Kaupichthysnuchalis* Böhlke, 1967	Collared Eel		2,11	1			
**Chlorophthalmidae**							
*Parasudistruculenta* (Goode & Bean, 1896)	Longnose Greeneye		5				
**Cichlidae**							
*Oreochromismossambicus* (Peters, 1852)	Mozambique Tilapia		6	1			
**Cirrhitidae**							
*Amblycirrhituspinos* (Mowbray, 1927)	Redspotted Hawkfish	S5	2,4	1		1	
**Clupeidae**							
*Harengulaclupeola* (Cuvier, 1829)	False Pilchard		2	1			YES
*Harengulahumeralis* (Cuvier, 1829)	Redear Sardine	S5	2	1	SJ28, SJ13		YES
*Harengulajaguana* Poey, 1865	Scaled Sardine			FlMNH			
*Opisthonemaoglinum* (Lesueur, 1818)	Atlantic Thread Herring			FlMNH			YES
*Sardinellaaurita* Valenciennes, 1847	Spanish Sardine			FlMNH			
**Congridae**							
*Ariosomabalearicum* (Delaroche, 1809)	Bandtooth Conger		2				
*Congertriporiceps* Kanazawa, 1958	Manytooth Conger		4	1			
*Heterocongerlongissimus* Günther, 1870	Brown Garden Eel	S5	2,4	1			
*Xenomystaxbidentatus* (Reid, 1940)	Rabbit Conger			NMNH			
**Coryphaenidae**							
*Coryphaenaequiselis* Linnaeus, 1758	Pompano Dolphinfish			ROM			
*Coryphaenahippurus* Linnaeus, 1758	Dolphinfish	S5	2	1			
**Cynoglossidae**							
*Symphurusarawak* Robins & Randall, 1965	Caribbean Tonguefish		2	1		1	
**Dactylopteridae**							
*Dactylopterusvolitans* (Linnaeus, 1758)	Flying Gurnard	S5	4	1			YES
**Dactyloscopidae**							
*Dactyloscopuscomptus* Dawson, 1982	Ornamented Stargazer		2,11	1			
*Dactyloscopuscrossotus* Starks, 1913	Bigeye Stargazer			AMNH			
*Dactyloscopuspoeyi* Gill, 1861	Shortchin Stargazer			FlMNH			
*Dactyloscopustridigitatus* Gill, 1859	Sand Stargazer	S5	2	1		1	
*Gillellusgreyae* Kanazawa, 1952	Arrow Stargazer		2			-1	
*Gillellusuranidea* Böhlke, 1968	Warteye Stargazer		2			-1	YES
*Platygillellusrubrocinctus* (Longley, 1934)	Saddle Stargazer						
**Dasyatidae**							
*Hypanusamericanus* (Hildebrand & Schroeder, 1928)	Southern Stingray	S5	1,2,4,10	1			
**Diodontidae**							
*Chilomycterusantennatus* (Cuvier, 1816)	Bridled Burrfish	S5	2,4	1	SJ18		
*Chilomycterusantillarum* Jordan & Rutter, 1897	Web Burrfish		2	1			
*Diodonholocanthus* Linnaeus, 1758	Balloonfish	S5	2,4	1	SJ11, SJ13	-1	
*Diodonhystrix* Linnaeus, 1758	Porcupinefish	S5	2,4	1		1	
**Echeneidae**							
*Echeneisnaucrates* Linnaeus, 1758	Sharksucker	S6	2,4	1	SJ19, SJ23		YES
*Echeneisneucratoides* Zuiew, 1789	Whitefin Sharksucker	S6		1			
*Remoraremora* (Linnaeus, 1758)	The Remora	S6		1	O3		YES
**Eleotridae**							
*Dormitatormaculatus* (Bloch, 1792)	Fat Sleeper	S6	6	1	SJ10		
*Eleotrisperniger* (Cope, 1871)	Smallscaled Spinycheek Sleeper	S6	6	1	SJ10		
*Erotelissmaragdus* (Valenciennes, 1837)	Emerald Sleeper		6	1			
*Gobiomorusdormitor* Lacepède, 1800	Bigmouth Sleeper	S6		1			
**Elopidae**							
*Elopssmithi* McBride, Rocha, Ruiz-Carus & Bowen, 2010	Malacho		2,6				YES
**Engraulidae**							
*Anchoalyolepis* (Evermann & Marsh, 1900)	Dusky Anchovy		2	1			YES
**Ephippidae**							
*Chaetodipterusfaber* (Broussonet, 1782)	Atlantic Spadefish	S6	2,4	1	SJ18, ST2		
**Epigonidae**							
*Epigonuspandionis* (Goode & Bean, 1881)	Caudal-ring Deepwater Cardinalfish			CAS			
**Exocoetidae**							
*Cheilopogonexsiliens* (Linnaeus, 1771)	Bandwing Flyingfish		2	1			
*Exocoetusobtusirostris* Günther, 1866	Oceanic Two-wing Flyingfish			MCZ			
*Hirundichthysaffinis* (Günther, 1866)	Fourwing Flyingfish		2				
*Hirundichthysspeculiger* (Valenciennes, 1847)	Mirrorwing Flyingfish		2	1			
*Prognichthysoccidentalis* Parin, 1999	Bluntnose Flyingfish	S6					YES
**Fistulariidae**							
*Fistulariatabacaria* Linnaeus, 1758	Bluespotted Cornetfish	S6	2		O4		
**Galeocerdonidae**							
*Galeocerdocuvier* (Peron & Lesueur, 1822)	Tiger Shark		10				
**Gempylidae**							
*Epinnulamagistralis* Poey, 1854	Domine		5	1			
**Gerreidae**							
*Eucinostomusargenteus* Baird & Girard, 1855	Spotfin Mojarra		2	1			YES
*Eucinostomusgula* (Quoy & Gaimard, 1824)	Silver Jenny	S6	4	1	SJ18, SJ13, SJ3		
*Eucinostomusharengulus* Goode & Bean, 1879	Tidewater Mojarra	S6		1	SJ28		
*Eucinostomushavana* (Nichols, 1912)	Bigeye Mojarra			FlMNH			
*Eucinostomusjonesii* (Günther, 1879)	Slender Mojarra	S6	4,6		SJ28		
*Eucinostomuslefroyi* (Goode, 1874)	Mottled Mojarra	S6			SJ28, SJ21		
*Eucinostomusmelanopterus* (Bleeker, 1863)	Flagfin Mojarra	S6	4	1	SJ28		
*Eugerresbrasilianus* (Cuvier, 1830)	Brazilian Mojarra		6,11	1			
*Gerrescinereus* (Walbaum, 1792)	Yellowfin Mojarra	S6	2,4,6	1			
**Ginglymostomatidae**							
*Ginglymostomacirratum* (Bonnaterre, 1788)	Nurse Shark	S6	1,2,4,10	1			
**Gobiesocidae**							
*Acyrtopsamplicirrus* Briggs, 1955	Flarenostril Clingfish		2				
*Acyrtopsberyllinus* (Hildebrand & Ginsburg, 1927)	Emerald Clingfish		2	1			
*Acyrtusartius* Briggs, 1955	Papillate Clingfish		2				
*Acyrtusrubiginosus* (Poey, 1868)	Red Clingfish	S6		1	SJ23, SJ13, SJ5		YES
*Arcosnudus* (Linnaeus, 1758)	Padded Clingfish	S6	2	1	SJ23	1	
*Gobiesoxnigripinnis* (Peters, 1859)	Dark-finned Clingfish	S6	2	1	SJ29		
*Gobiesoxpunctulatus* (Poey, 1876)	Stippled Clingfish	S6	2	1		1	YES
*Tomicodoncryptus* Williams & Tyler, 2003	Cryptic Clingfish	S6					YES
*Tomicodonfasciatus* (Peters, 1859)	Barred Clingfish		2	1		1	
*Tomicodonleurodiscus* Williams & Tyler, 2003	Smooth-suckered Clingfish		11	1			
*Tomicodonreitzae* Briggs, 2001	Accidental Clingfish	S6					YES
*Tomicodonrhabdotus* Smith-Vaniz, 1969	Antillean Clingfish	S6			O24		
*Tomicodonrupestris* (Poey, 1860)	Barred Clingfish		11	1			
**Gobiidae**							
*Awaousbanana* (Valenciennes, 1837)	River Goby	S7		1	SJ10		
*Barbuliferceuthoecus* (Jordan & Gilbert, 1884)	Bearded Goby		2	1			YES
*Bathygobiusantilliensis* Tornabene, Baldwin & Pezold, 2010	Antilles Frillfin	S7			SJ36		YES
*Bathygobiuscuracao* (Metzelaar, 1919)	Notchtongue Goby		11	1			YES
*Bathygobiuslacertus* (Poey, 1860)	Checkerboard Frillfin			FlMNH			YES
*Bathygobiusmystacium* Ginsburg, 1947	Island Frillfin	S7			SJ21, SJ19		YES
*Bathygobiussoporator* (Valenciennes, 1837)	Frillfin Goby		2,6,11	1			YES
*Bollmanniaboqueronensis* Evermann & Marsh, 1899	White-eye Goby	S7	4		SJ19		YES
*Cerdalefloridana* Longley, 1934	Pugjaw Wormfish	S7	2	1	SJ23	1	
*Coryphopterusalloides* Böhlke & Robins, 1960	Barfin Goby		2	1		-1	
*Coryphopterusdicrus* Böhlke & Robins, 1960	Colon Goby	S7	2,4	1		1	YES
*Coryphopteruseidolon* Böhlke & Robins, 1960	Pallid Goby	S7	2,4	1		1	YES
*Coryphopterusglaucofraenum* Gill, 1863	Bridled Goby	S7	2,4	1		1	YES
*Coryphopterushyalinus* Böhlke & Robins, 1962	Glass Goby	S7	2	1		-1	YES
*Coryphopteruskuna* Victor, 2007	Kuna Goby	S7			SJ5, SJ12		
*Coryphopteruslipernes* Böhlke & Robins, 1962	Peppermint Goby	S7	2,4	1	ST6		YES
*Coryphopteruspersonatus* (Jordan & Thompson, 1905)	Masked Goby	S7	2	1		1	YES
*Coryphopterusthrix* Böhlke & Robins, 1960	Bartail Goby		2	1		1	YES
*Coryphopterustortugae* (Jordan, 1904)	Sand Goby	S7		1			YES
*Coryphopterusvenezuelae* Cervigón, 1966	Venezuela Goby	S7		1			YES
*Ctenogobiusboleosoma* (Jordan & Gilbert, 1882)	Darter Goby	S7	6	1	SJ28		YES
*Ctenogobiussaepepallens* (Gilbert & Randall, 1968)	Dash Goby	S7	2,4	1			YES
*Ctenogobiussmaragdus* (Valenciennes, 1837)	Emerald Goby		11				
*Ctenogobiusstigmaturus* (Goode & Bean, 1882)	Spottail Goby	S7			SJ28		YES
*Elacatinuschancei* (Beebe & Hollister, 1933)	Shortstripe Goby	S7	2,4	1			YES
*Elacatinusevelynae* (Böhlke & Robins, 1968)	Sharknose Goby	S7	2,4	1		1	YES
*Elacatinusprochilos* (Böhlke & Robins, 1968)	Broadstripe Goby	S7		1			YES
*Evorthoduslyricus* (Girard, 1858)	Lyre Goby		6	1			
*Ginsburgellusnovemlineatus* (Fowler, 1950)	Ninelined Goby	S7		1	SJ23, SJ5		YES
*Gnatholepisthompsoni* Jordan, 1904	Goldspot Goby	S7	2,4	1		1	YES
*Gobionellusoceanicus* (Pallas, 1770)	Highfin Goby	S7		1	SJ28		
*Gobiosomagrosvenori* (Robins, 1964)	Rockcut Goby		4	1			
*Lophogobiuscyprinoides* (Pallas, 1770)	Crested Goby	S8	6	1	SJ28		
*Lythrypnuselasson* Böhlke & Robins, 1960	Dwarf Goby	S8	2	AMNH	ST5	1	YES
*Lythrypnusminimus* Garzón-Ferreira & Acero P., 1988	Pygmy Goby	S8					YES
*Lythrypnusnesiotes* Böhlke & Robins, 1960	Island Goby	S8	2	1	SJ34	1	YES
*Lythrypnusspilus* Böhlke & Robins, 1960	Bluegold Goby	S8			ST3		
*Microgobiuscarri* Fowler, 1945	Seminole Goby	S8	2,4	1	SJ19, SJ25	1	YES
*Microgobiussignatus* Poey, 1876	Signal Goby	S8		1	SJ28, SJ22, SJ3		YES
*Neslongus* (Nichols, 1914)	Orangespotted Goby	S8	4	1			YES
*Oxyurichthysstigmalophius* (Mead & Böhlke, 1958)	Spotfin Goby	S8	4	1	SJ5, SJ19, SJ28		
*Palatogobiusparadoxus* Gilbert, 1971	Mauve Goby		2,11	1			
*Priolepishipoliti* (Metzelaar, 1922)	Rusty Goby	S8	2,4	1		1	
*Psilotriscelsa* Böhlke, 1963	Highspine Goby		2	1			
*Ptereleotrishelenae* (Randall, 1968)	Hovering Dartfish	S8	2,4	1			
*Risorruber* (Rosén, 1911)	Tusked Goby	S8	2	1		1	YES
*Sicydiumplumieri* (Bloch, 1786)	Sirajo Goby	S8	6	1	SJ10		YES
*Sicydiumpunctatum* Perugia, 1896	Spotted Algae-eating Goby	S8		1	SJ10		YES
*Tigrigobiusdilepis* (Robins & Böhlke, 1964)	Orangesided Goby		4	1			
*Tigrigobiusmultifasciatus* (Steindachner, 1876)	Greenbanded Goby	S8	2	1			YES
*Tigrigobiuspallens* (Ginsburg, 1939)	Semiscaled Goby	S8			SJ23		
*Tigrigobiussaucrus* (Robins, 1960)	Leopard Goby	S8	2	1		1	YES
**Grammatidae**							
*Grammalinki* Starck & Colin, 1978	Yellowcheek Basslet		2,5,8			1	
*Grammaloreto* Poey, 1868	Fairy Basslet	S8	2,4	1			YES
**Haemulidae**							
*Anisotremussurinamensis* (Bloch, 1791)	Black Margate	S9	2,4,5,8	1		1	YES
*Anisotremusvirginicus* (Linnaeus, 1758)	Porkfish	S9	2,5,6,8	1			YES
*Brachygenyschrysargyrea* (Günther, 1859)	Smallmouth Grunt	S9	2,4	1		1	YES
*Emmelichthyopsatlanticus* Schultz, 1945	Bonnetmouth	S9	2		ST8		
*Haemulonalbum* Cuvier, 1830	Margate	S9	2,4	1	SJ7		
*Haemulonaurolineatum* Cuvier, 1830	Tomtate	S9	2,4,5,8	1		1	YES
*Haemuloncarbonarium* Poey, 1860	Caesar Grunt	S9	2,4	1		1	
*Haemulonflavolineatum* (Desmarest, 1823)	French Grunt	S9	2,4,5,8	1		1	YES
*Haemulonmacrostoma* Günther, 1859	Spanish Grunt	S9	2,4	1		1	
*Haemulonmelanurum* (Linnaeus, 1758)	Cottonwick	S9	2	1	O5		YES
*Haemulonparra* (Desmarest, 1823)	Sailors Choice	S9	2,4	1	SJ1, SJ21		YES
*Haemulonplumierii* (Lacepède, 1801)	White Grunt	S9	2,4	1		1	YES
*Haemulonsciurus* (Shaw, 1803)	Bluestriped Grunt	S9	2,4,5	1		1	YES
*Haemulonstriatum* (Linnaeus, 1758)	Striped Grunt		2,4	1			YES
*Haemulonvittatum* (Poey, 1860)	Boga	S9	2,4	1	ST6, ST8, ST2	1	
**Hemiramphidae**							
*Euleptorhamphusvelox* Poey, 1868	Flying Halfbeak			MCZ			
*Hemiramphusbalao* Lesueur, 1821	Balao			MCZ			
*Hemiramphusbrasiliensis* (Linnaeus, 1758)	Ballyhoo	S9	2	1			
*Hyporhamphusunifasciatus* (Ranzani, 1841)	Atlantic Silverstripe Halfbeak		2	1			
**Hexanchidae**							
*Heptranchiasperlo* (Bonnaterre, 1788)	Sharpnose Sevengill Shark			FlMNH			
*Hexanchusvitulus* Springer & Waller, 1969	Atlantic Sixgill Shark			FlMNH			
**Holocentridae**							
*Holocentrusadscensionis* (Osbeck, 1765)	Squirrelfish	S9	2,4,5,8	1		1	YES
*Holocentrusrufus* (Walbaum, 1792)	Longspine Squirrelfish	S9	2,4,5,8	1		1	
*Myripristisjacobus* Cuvier, 1829	Blackbar Soldierfish	S9	2,4,5,8	1		1	
*Neoniphoncoruscum* (Poey, 1860)	Reef Squirrelfish	S9	2,4,5,8	1		1	YES
*Neoniphonmarianus* (Cuvier, 1829)	Longjaw Squirrelfish	S9	2,4,5,8	1		1	
*Neoniphonvexillarium* (Poey, 1860)	Dusky Squirrelfish	S9	2,4	1		1	
*Ostichthystrachypoma* (Günther, 1859)	Bigeye Soldierfish		2,5,8	1			
*Plectrypopsretrospinis* (Guichenot, 1853)	Cardinal Soldierfish	S9	2,5,8	1	SJ9, SJ22, ST3	1	
*Sargocentronbullisi* (Woods, 1955)	Deepwater Squirrelfish		2,11	1			
**Ipnopidae**							
*Bathypteroisbigelowi* Mead, 1958	Spottail Tripodfish			CAS			
*Bathypteroisphenax* Parr, 1928	Blackfin Spiderfish		9				
*Bathypteroisviridensis* (Roule, 1916)	Twobanded Tripodfish		9				
*Ipnopsmurrayi* Günther, 1878	Grideye Fish		9				
**Istiophoridae**							
*Istiophorusplatypterus* (Shaw, 1792)	Sailfish	S9	2				
*Kajikiaalbida* (Poey, 1860)	White Marlin	S9	2				
*Makairanigricans* Lacepède, 1802	Blue Marlin		2				YES
*Tetrapturuspfluegeri* Robins & de Sylva, 1963	Longbill Spearfish		2				
**Kyphosidae**							
*Kyphosuscinerascens* (Forsskål, 1775)	Topsail Seachub	S10					
*Kyphosussectatrix* (Linnaeus, 1758)	Bermuda Chub	S10	2,4	1			
*Kyphosusvaigiensis* (Quoy & Gaimard, 1825)	Yellow Chub	S10		1			
**Labridae**							
**Labrinae**							
*Bodianusrufus* (Linnaeus, 1758)	Spanish Hogfish	S10	2,4,5,8	1			YES
*Clepticusparrae* (Bloch & Schneider, 1801)	Creole Wrasse	S10	2,4,5,8	1			YES
*Decodonpuellaris* (Poey, 1860)	Red Hogfish		2	1			
*Doratonotusmegalepis* Günther, 1862	Dwarf Wrasse		2	1			
*Halichoeresbivittatus* (Bloch, 1791)	Slippery Dick	S10	2,4	1		1	YES
*Halichoerescaudalis* (Poey, 1860)	Painted Wrasse			NOAA			
*Halichoerescyanocephalus* (Bloch, 1791)	Yellowcheek Wrasse		2	1			
*Halichoeresgarnoti* (Valenciennes, 1839)	Yellowhead Wrasse	S10	2,4	1		1	YES
*Halichoeresmaculipinna* (Müller & Troschel, 1848)	Clown Wrasse	S10	2,4	1		1	
*Halichoerespictus* (Poey, 1860)	Rainbow Wrasse	S10	2,4	1		1	
*Halichoerespoeyi* (Steindachner, 1867)	Blackear Wrasse	S10	2,4	1			
*Halichoeresradiatus* (Linnaeus, 1758)	Puddingwife	S10	2,4	1		1	YES
*Lachnolaimusmaximus* (Walbaum, 1792)	Hogfish	S10	2,4,5,8	1			
*Thalassomabifasciatum* (Bloch, 1791)	Bluehead	S10	2,4	1		1	
*Xyrichtysmartinicensis* Valenciennes, 1840	Rosy Razorfish	S10	2,4	1			
*Xyrichtysnovacula* (Linnaeus, 1758)	Pearly Razorfish	S10	2,4	1			YES
*Xyrichtyssplendens* Castelnau, 1855	Green Razorfish	S10	2,4	1			
**Scarinae**							
*Cryptotomusroseus* Cope, 1871	Bluelip Parrotfish	S10	2,4	1			YES
*Scaruscoelestinus* Valenciennes, 1840	Midnight Parrotfish	S10	2	1	O6	1	
*Scaruscoeruleus* (Edwards, 1771)	Blue Parrotfish		2,4	1		1	
*Scarusguacamaia* Cuvier, 1829	Rainbow Parrotfish	S10	2,4	1	SJ28, SJ33, O2		
*Scarusiseri* (Bloch, 1789)	Striped Parrotfish	S10	2,4	1		1	YES
*Scarustaeniopterus* Lesson, 1829	Princess Parrotfish	S10	2,4,5,8	1		1	YES
*Scarusvetula* Bloch & Schneider, 1801	Queen Parrotfish	S10	2,4	1		1	YES
*Sparisomaatomarium* (Poey, 1861)	Greenblotch Parrotfish	S11	2,4	1			
*Sparisomaaurofrenatum* (Valenciennes, 1840)	Redband Parrotfish	S11	2,4,5,8	1		1	YES
*Sparisomachrysopterum* (Bloch & Schneider, 1801)	Redtail Parrotfish	S11	2,4	1		1	YES
*Sparisomaradians* (Valenciennes, 1840)	Bucktooth Parrotfish	S11	2,4	1		1	YES
*Sparisomarubripinne* (Valenciennes, 1840)	Yellowtail Parrotfish	S11	2,4	1		1	YES
*Sparisomaviride* (Bonnaterre, 1788)	Stoplight Parrotfish	S11	2,4,5,8	1		1	YES
**Labrisomidae**							
*Brockiusalbigenys* Beebe & Tee-Van, 1928	Whitecheek Blenny		DNA		Berry Bay, St. John		YES
*Brockiusnigricinctus* (Howell Rivero, 1936)	Spotcheek Blenny	S11		1	SJ21		YES
*Gobioclinusbucciferus* (Poey, 1868)	Puffcheek Blenny	S11	2	1			YES
*Gobioclinusfilamentosus* (Springer, 1960)	Quillfin Blenny	S11	3,11	1	O7		YES
*Gobioclinusgobio* (Valenciennes, 1836)	Palehead Blenny	S11	2	1		1	YES
*Gobioclinusguppyi* (Norman, 1922)	Mimic Blenny	S11	2	1		-1	YES
*Gobioclinushaitiensis* (Beebe & Tee-Van, 1928)	Longfin Blenny	S11	2	1	SJ12	1	YES
*Labrisomuscricota* Sazima, Gasparini & Moura, 2002	Mock Blenny	S11			SJ10		
*Labrisomusnuchipinnis* (Quoy & Gaimard, 1824)	Hairy Blenny	S11	2,4	1		1	YES
*Malacoctenusaurolineatus* Smith, 1957	Goldline Blenny	S11	2,4	1		1	YES
*Malacoctenusboehlkei* Springer, 1959	Diamond Blenny	S11	2,4	1		1	YES
*Malacoctenuserdmani* Smith, 1957	Imitator Blenny	S11			SJ23		YES
*Malacoctenusgilli* (Steindachner, 1867)	Dusky Blenny	S11	2,4	1			YES
*Malacoctenusmacropus* (Poey, 1868)	Rosy Blenny	S11	2,4	1			YES
*Malacoctenustriangulatus* Springer, 1959	Saddled Blenny	S11	2,4	1		1	YES
*Malacoctenusversicolor* (Poey, 1876)	Barfin Blenny	S11	2,4	1	SJ23, SJ12		YES
*Nemaclinusatelestos* Böhlke & Springer, 1975	Threadfin Blenny		2,11	1			
*Paraclinusbarbatus* Springer, 1955	Goatee Blenny		2,11				
*Paraclinuscingulatus* (Evermann & Marsh, 1899)	Coral Blenny		2				
*Paraclinusfasciatus* (Steindachner, 1876)	Banded Blenny	S11	2		SJ12		
*Paraclinusnigripinnis* (Steindachner, 1867)	Blackfin Blenny	S11	2	1	SJ12		YES
*Starksiaculebrae* (Evermann & Marsh, 1899)	Culebra Blenny	S11	2	1	ST2, SJ13		YES
*Starksiahassi* Klausewitz, 1958	Ringed Blenny	S11	2,11	1	SJ24	1	
*Starksialepicoelia* Böhlke & Springer, 1961	Blackcheek Blenny		2	1		1	
*Starksiananodes* Böhlke & Springer, 1961	Dwarf Blenny		2	1			
*Starksiawilliamsi* Baldwin & Castillo, 2011	Williams’s Blenny	S11			SJ2, SJ13		YES
*Stathmonotusgymnodermis* Springer, 1955	Naked Blenny		2	1			
*Stathmonotusstahli* (Evermann & Marsh, 1899)	Southern Eelgrass Blenny		2	1			
**Latilidae**							
*Caulolatiluscyanops* Poey, 1866	Blackline Tilefish		2				
**Lobotidae**							
*Lobotessurinamensis* (Bloch, 1790)	Atlantic Tripletail	S11	2	1	O18		
**Lutjanidae**							
*Apsilusdentatus* Guichenot, 1853	Black Snapper		2				
*Etelisoculatus* (Valenciennes, 1828)	Queen Snapper	S12	2,5,8				YES
*Lutjanusanalis* (Cuvier, 1828)	Mutton Snapper	S12	2,4,5,8	1			YES
*Lutjanusapodus* (Walbaum, 1792)	Schoolmaster	S12	2,4,5,6,8	1		1	YES
*Lutjanusbuccanella* (Cuvier, 1828)	Blackfin Snapper	S12	2,5,8	1			YES
*Lutjanuscyanopterus* (Cuvier, 1828)	Cubera Snapper	S12	2,4	1			YES
*Lutjanusgriseus* (Linnaeus, 1758)	Gray Snapper	S12	2,4,6	1		1	
*Lutjanusjocu* (Bloch & Schneider, 1801)	Dog Snapper	S12	2,4,5,8	1			YES
*Lutjanusmahogoni* (Cuvier, 1828)	Mahogany Snapper	S12	2,4	1		1	YES
*Lutjanuspurpureus* (Poey, 1866)	Caribbean Red Snapper		2	1			
*Lutjanussynagris* (Linnaeus, 1758)	Lane Snapper	S12	2,4	1			YES
*Lutjanusvivanus* (Cuvier, 1828)	Silk Snapper	S12	2,5,8	1			YES
*Ocyuruschrysurus* (Bloch, 1791)	Yellowtail Snapper	S12	2,4	1		1	YES
*Pristipomoidesmacrophthalmus* (Müller & Troschel, 1848)	Cardinal Snapper		2				
*Rhomboplitesaurorubens* (Cuvier, 1829)	Vermilion Snapper	S12	2		SJ20		
**Malacanthidae**							
*Malacanthusplumieri* (Bloch, 1786)	Sand Tilefish	S12	2,4,5,8	1		1	
**Megalopidae**							
*Megalopsatlanticus* Valenciennes, 1847	Tarpon	S12	2,6	1			
**Mobulidae**							
*Mobulabirostris* (Walbaum, 1792)	Giant Manta	S12	2				
*Mobulacfbirostris*	Caribbean Manta	S12			SJ12		
**Monacanthidae**							
*Aluterusmonoceros* (Linnaeus, 1758)	Unicorn Filefish	S12			O23		
*Aluterusschoepfii* (Walbaum, 1792)	Orange Filefish	S12		1	SJ34		
*Aluterusscriptus* (Osbeck, 1765)	Scrawled Filefish	S12	4	1			
*Cantherhinesmacrocerus* (Hollard, 1853)	Whitespotted Filefish	S12	2	1			YES
*Cantherhinespullus* (Ranzani, 1842)	Orangespotted Filefish	S12	2,4	1		1	
*Monacanthusciliatus* (Mitchill, 1818)	Fringed Filefish	S12	2,4	1			YES
*Monacanthustuckeri* Bean, 1906	Slender Filefish	S12	2,4	1			
*Stephanolepishispida* (Linnaeus, 1766)	Planehead Filefish			FlMNH			
*Stephanolepissetifer* (Bennett, 1831)	Pygmy Filefish		2				
**Moringuidae**							
*Moringuaedwardsi* (Jordan & Bollman, 1889)	Spaghetti Eel		2	1		-1	
**Mugilidae**							
*Dajausmonticola* (Bancroft, 1834)	Mountain Mullet	S13	6	1	SJ10		
*Mugilcurema* Valenciennes, 1836	White Mullet	S13	2,6		SJ21		YES
*Mugilrubrioculus* Harrison, Nirchio, Oliveira, Ron & Gaviria, 2007	Redeye Mullet	S13	DNA				YES
*Mugiltrichodon* Poey, 1875	Fantail Mullet			ROM			
**Mullidae**							
*Mulloidichthysmartinicus* (Cuvier, 1829)	Yellow Goatfish	S13	2,4,6	1		1	YES
*Pseudupeneusmaculatus* (Bloch, 1793)	Spotted Goatfish	S13	2,4,5,8	1		1	
**Muraenidae**							
*Echidnacatenata* (Bloch, 1795)	Chain Moray	S13	2,4	1	SJ21, SJ10	1	
*Enchelycorecarychroa* Böhlke & Böhlke, 1976	Chestnut Moray	S13	2	1	SJ5	1	
*Enchelycorenigricans* (Bonnaterre, 1788)	Viper Moray	S13	2	1	SJ9	1	
*Gymnothoraxconspersus* Poey, 1867	Saddled Moray			ANSP			
*Gymnothoraxfunebris* Ranzani, 1839	Green Moray	S13	2,4	1		1	YES
*Gymnothoraxmaderensis* (Johnson, 1862)	Sharktooth Moray		2	1			
*Gymnothoraxmiliaris* (Kaup, 1856)	Goldentail Moray	S13	2,4	1		1	
*Gymnothoraxmoringa* (Cuvier, 1829)	Spotted Moray	S13	2,4			1	YES
*Gymnothoraxvicinus* (Castelnau, 1855)	Purplemouth Moray	S13	2,4	1		1	
*Uropterygiusmacularius* (Lesueur, 1825)	Marbled Moray	S13	2	1		1	
**Myctophidae**							
*Centrobranchusnigroocellatus* (Günther, 1873)	Roundnose Lanternfish			ROM			
**Neoscopelidae**							
*Neoscopelusmacrolepidotus* Johnson, 1863	Largescale Blackchin			CAS			
**Nomeidae**							
*Psenescyanophrys* Valenciennes, 1833	Freckled Driftfish		2	1			
**Ogcocephalidae**							
*Ogcocephalusnasutus* (Cuvier, 1829)	Shortnose Batfish		2	1			
*Ogcocephaluspumilus* Bradbury, 1980	Dwarf Batfish			CAS			
**Ophichthidae**							
*Ahliaegmontis* (Jordan, 1884)	Key Worm Eel		2	1			
*Aprognathodonplatyventris* Böhlke, 1967	Stripe Eel		2	1			
*Callechelysguineensis* (Osório, 1893)	Shorttail Snake Eel		11	1			
*Echiophisintertinctus* (Richardson, 1848)	Spotted Spoon-nose Eel		2				
*Ichthyapusophioneus* (Evermann & Marsh, 1900)	Surf Eel			FlMNH			
*Myrichthysbreviceps* (Richardson, 1848)	Sharptail Eel	S13	2		SJ13		
*Myrichthysocellatus* (Lesueur, 1825)	Goldspotted Eel		2	1			
*Myrophisanterodorsalis* McCosker, Böhlke & Böhlke, 1989	Longfin Worm Eel	S13			SJ28		
*Myrophisplatyrhynchus* Breder, 1927	Broadnose Worm Eel		2	1			YES
*Myrophispunctatus* Lütken, 1852	Speckled Worm Eel		2,11	1			
**Ophidiidae**							
*Brotulabarbata* (Bloch & Schneider, 1801)	Atlantic Bearded Brotula		2	1		-1	
*Lepophidiumpheromystax* Robins, 1960	Upsilon Cusk-eel		2	1			
Luciobrotulacorethromycter* Cohen, 1964	Broomnose Cusk-eel		9				
*Ophidionholbrookii* Putnam, 1874	Bank Cusk-eel		2,3,11	1		-1	
*Parophidionschmidti* (Woods & Kanazawa, 1951)	Dusky Cusk-eel			1			
Xyelacybamyersi* Cohen, 1961	Gargoyle Cusk-eel		9				
**Opistognathidae**							
*Lonchopisthusmicrognathus* (Poey, 1860)	Swordtail Jawfish	S13	4	1	SJ28, SJ19		YES
*Opistognathusaurifrons* (Jordan & Thompson, 1905)	Yellowhead Jawfish	S13	2,4	1			YES
*Opistognathusmacrognathus* Poey, 1860	Banded Jawfish	S13	2,4,11	1	SJ5, SJ13, SJ19		
*Opistognathusmaxillosus* Poey, 1860	Mottled Jawfish	S13	2	1	SJ5, SJ13, SJ19	1	
*Opistognathuswhitehursti* (Longley, 1927)	Dusky Jawfish	S13		1	SJ12		
**Ostraciidae**							
*Acanthostracionpolygonium* Poey, 1876	Honeycomb Cowfish	S13	2	1			
*Acanthostracionquadricornis* (Linnaeus, 1758)	Scrawled Cowfish	S13	2	1			
*Lactophrysbicaudalis* (Linnaeus, 1758)	Spotted Trunkfish	S13	2,4	1			
*Lactophrystrigonus* (Linnaeus, 1758)	Trunkfish	S13	2,4	1			
*Lactophrystriqueter* (Linnaeus, 1758)	Smooth Trunkfish	S13	2,4	1		1	
**Paralichthyidae**							
*Citharichthyscornutus* (Günther, 1880)	Horned Whiff			FMNH			
*Citharichthysuhleri* Jordan, 1889	Voodoo Whiff			FlMNH			
*Cyclopsettafimbriata* (Goode & Bean, 1885)	Spotfin Flounder	S14	2	1	SJ12, O14		
*Syaciummicrurum* Ranzani, 1842	Channel Flounder		2	1			YES
**Parazenidae**							
*Cyttopsisrosea* (Lowe, 1843)	Red Dory		5				
**Pempheridae**							
*Pempherisschomburgkii* Müller & Troschel, 1848	Glassy Sweeper	S14	2,4	1	SJ13, ST3, SJ15		YES
**Poeciliidae**							
*Poeciliareticulata* Peters, 1859	Guppy	S14		1	SJ10		
**Polymixiidae**							
*Polymixialowei* Günther, 1859	Beardfish			FlMNH, CAS			
*Polymixianobilis* Lowe, 1836	Noble Beardfish		5,8				
**Polynemidae**							
*Polydactylusvirginicus* (Linnaeus, 1758)	Barbu			FlMNH			
**Pomacanthidae**							
*Centropygeargi* Woods & Kanazawa, 1951	Cherubfish	S14	2,4,5,8	1	O21		
*Holacanthusciliaris* (Linnaeus, 1758)	Queen Angelfish	S14	2,4	1		1	YES
*Holacanthustricolor* (Bloch, 1795)	Rock Beauty	S14	2,4,5,8	1		1	
*Pomacanthusarcuatus* (Linnaeus, 1758)	Gray Angelfish	S14	2,4,5,8	1		1	
*Pomacanthusparu* (Bloch, 1787)	French Angelfish	S14	2,4,5	1		1	
**Pomacentridae**							
*Abudefdufsaxatilis* (Linnaeus, 1758)	Sergeant Major	S14	2,4,6	1		1	YES
*Abudefduftaurus* (Müller & Troschel, 1848)	Night Sergeant	S14	2,4	1		1	
*Azurinacyanea* (Poey, 1860)	Blue Chromis	S14	2,4,8	1		1	YES
*Azurinamultilineata* (Guichenot, 1853)	Brown Chromis	S14	2,4,5,8	1		1	YES
*Chromisinsolata* (Cuvier, 1830)	Sunshinefish	S14	2,5,8	1	O20		
*Microspathodonchrysurus* (Cuvier, 1830)	Yellowtail Damselfish	S14	2,4,5	1		1	
*Stegastesadustus* (Troschel, 1865)	Dusky Damselfish	S14	2,4,6	1		1	
*Stegastesdiencaeus* (Jordan & Rutter, 1897)	Longfin Damselfish	S14	2,4	1			YES
*Stegastesleucostictus* (Müller & Troschel, 1848)	Beaugregory	S14	2,4	1		1	YES
*Stegastespartitus* (Poey, 1868)	Bicolor Damselfish	S14	2,4,5,8	1		1	YES
*Stegastesplanifrons* (Cuvier, 1830)	Threespot Damselfish	S14	2,4	1		1	YES
*Stegastesxanthurus* (Poey, 1860)	Cocoa Damselfish	S14	2,4	1		1	YES
**Priacanthidae**							
*Heteropriacanthuscruentatus* (Lacepède, 1801)	Glasseye Snapper	S15	2,4	1		1	YES
*Priacanthusarenatus* Cuvier, 1829	Bigeye	S15	2	1	SJ24		
*Pristigenysalta* (Gill, 1862)	Short Bigeye		2	1			
**Rachycentridae**							
*Rachycentroncanadum* (Linnaeus, 1766)	Cobia	S15			ST3		
**Rhincodontidae**							
*Rhincodontypus* Smith, 1828	Whale Shark	S15					
**Rivulidae**							
*Kryptolebiasmarmoratus* (Poey, 1880)	Mangrove Rivulus		6	1			
**Sciaenidae**							
*Corvulabatabana* (Poey, 1860)	Blue Croaker		2,11	1			
*Equeslanceolatus* (Linnaeus, 1758)	Jackknife-fish	S15	2,4	1	SJ30		
*Equespunctatus* Bloch & Schneider, 1801	Spotted Drum	S15	2,4	1		1	
*Odontosciondentex* (Cuvier, 1830)	Reef Croaker	S15	2,4	1		1	
*Parequesacuminatus* (Bloch & Schneider, 1801)	High-hat	S15	2,4	1		1	YES
*Umbrinacoroides* Cuvier, 1830	Sand Drum		2	1			
**Scomberesocidae**							
*Scomberesoxsaurus* (Walbaum, 1792)	Atlantic Saury			KU			
**Scombridae**							
*Acanthocybiumsolandri* (Cuvier, 1832)	Wahoo	S15	2				
*Euthynnusalletteratus* (Rafinesque, 1810)	Little Tunny	S15	2	1			YES
*Katsuwonuspelamis* (Linnaeus, 1758)	Skipjack Tuna	S15	2				
*Scomberomorusbrasiliensis* Collette, Russo & Zavala-Camin, 1978	Serra		2	1			
*Scomberomoruscavalla* (Cuvier, 1829)	King Mackerel	S15	2		SJ4, ST6		
*Scomberomorusregalis* (Bloch, 1793)	Cero	S15	2,4	1			
*Thunnusalbacares* (Bonnaterre, 1788)	Yellowfin Tuna	S15	2				
*Thunnusatlanticus* (Lesson, 1831)	Blackfin Tuna	S15	2	1			
**Scorpaenidae**							
*Pontinuscastor* Poey, 1860	Longsnout Scorpionfish		2	1			
*Pteroisvolitans* (Linnaeus, 1758)	Red Lionfish	S15		1			YES
*Scorpaenaalbifimbria* Evermann & Marsh, 1900	Coral Scorpionfish	S15	2,11	1	O8		
*Scorpaenabergii* Evermann & Marsh, 1900	Goosehead Scorpionfish			FlMNH			
*Scorpaenabrasiliensis* Cuvier, 1829	Barbfish		2,11	1			
*Scorpaenacalcarata* Goode & Bean, 1882	Smoothhead Scorpionfish		2,11	1			
*Scorpaenagrandicornis* Cuvier, 1829	Plumed Scorpionfish		2,6	1			
*Scorpaenainermis* Cuvier, 1829	Mushroom Scorpionfish	S15	2	1	SJ5		
*Scorpaenaplumieri* Bloch, 1789	Spotted Scorpionfish	S15	2,4	1		1	
*Scorpaenodescaribbaeus* Meek & Hildebrand, 1928	Reef Scorpionfish	S15	2	1	SJ34, SJ23, SJ13	1	
**Serranidae**							
*Alphestesafer* (Bloch, 1793)	Mutton Hamlet	S16	2	1	SJ23	1	
*Bullisichthyscaribbaeus* Rivas, 1971	Pugnose Bass		5,8				
*Cephalopholiscruentata* (Lacepède, 1802)	Graysby	S16	2,4,5,8	1		1	YES
*Cephalopholisfulva* (Linnaeus, 1758)	Coney	S16	2,4,5,8	1		1	
*Diplectrumbivittatum* (Valenciennes, 1828)	Dwarf Sand Perch	S16	2	1		1	YES
*Diplectrumformosum* (Linnaeus, 1766)	Sand Perch		4	1			
*Epinephelusadscensionis* (Osbeck, 1765)	Rock Hind	S16	2,4	1	SJ22, SJ15	1	
*Epinephelusguttatus* (Linnaeus, 1758)	Red Hind	S16	2,4,5,8	1		1	
*Epinephelusitajara* (Lichtenstein, 1822)	Atlantic Goliath Grouper	S16	2				
*Epinephelusmorio* (Valenciennes, 1828)	Red Grouper	S16	2	1			
*Epinephelusstriatus* (Bloch, 1792)	Nassau Grouper	S16	2,4,5,8	1		1	YES
*Hypoplectrusaberrans* Poey, 1868	Yellowbelly Hamlet	S16	2,4	1		1	
*Hypoplectruschlorurus* (Cuvier, 1828)	Yellowtail Hamlet	S16	2,4,5,8	1			
*Hypoplectrusguttavarius* (Poey, 1852)	Shy Hamlet	S16	2,4	1	SJ19, ST6		
*Hypoplectrusindigo* (Poey, 1851)	Indigo Hamlet	S16	2,4	1			
*Hypoplectrusnigricans* (Poey, 1852)	Black Hamlet	S16	2,4	1		1	
*Hypoplectruspuella* (Cuvier, 1828)	Barred Hamlet	S16	2,4	1		1	
*Hypoplectrusunicolor* (Walbaum, 1792)	Butter Hamlet	S16	2,4	1		1	
*Hyporthodusmystacinus* (Poey, 1852)	Misty Grouper		2,8				
*Liopropomamowbrayi* Woods & Kanazawa, 1951	Cave Basslet		2,5				
*Liopropomarubre* Poey, 1861	Peppermint Basslet	S16	2,4	1	ST1, SJ9, SJ13	1	
*Mycteropercaacutirostris* (Valenciennes, 1828)	Western Comb Grouper		2	1			
*Mycteropercabonaci* (Poey, 1860)	Black Grouper	S17	2	1	SJ33, O9, O10		
*Mycteropercainterstitialis* (Poey, 1860)	Yellowmouth Grouper	S17	2,4,5	1	SJ7		YES
*Mycteropercatigris* (Valenciennes, 1833)	Tiger Grouper	S17	2,5,8	1	O11, O12, O13	1	
*Mycteropercavenenosa* (Linnaeus, 1758)	Yellowfin Grouper	S17	2,4,5,8	1		1	YES
*Paranthiasfurcifer* (Valenciennes, 1828)	Atlantic Creolefish	S17	2,5,8	1	SJ33		
*Pronotogrammusmartinicensis* (Guichenot, 1868)	Roughtongue Bass		5				
*Rypticusbistrispinus* (Mitchill, 1818)	Freckled Soapfish	S17			O14		
*Rypticuscarpenteri* Baldwin & Weigt, 2012	Slope Soapfish	S17					
*Rypticussaponaceus* (Bloch & Schneider, 1801)	Greater Soapfish	S17	2,4	1		1	
*Rypticussubbifrenatus* Gill, 1861	Spotted Soapfish		2	1		1	
*Schultzeabeta* (Hildebrand, 1940)	School Bass	S17	2	1	O19		YES
*Serraniculuspumilio* Ginsburg, 1952	Pygmy Sea Bass	S17	11	1	SJ19		YES
*Serranusannularis* (Günther, 1880)	Orangeback Bass	S17	2,11	1	O17		
*Serranusbaldwini* (Evermann & Marsh, 1899)	Lantern Bass	S17	2,4	1	SJ32, SJ12, SJ22		YES
*Serranusluciopercanus* Poey, 1852	Crosshatch Bass		2,5,8				
*Serranusphoebe* Poey, 1851	Tattler		2	1			
*Serranustabacarius* (Cuvier, 1829)	Tobaccofish	S17	2,4	1			YES
*Serranustigrinus* (Bloch, 1790)	Harlequin Bass	S17	2,4	1		1	
*Serranustortugarum* Longley, 1935	Chalk Bass	S17	2,4,5	1			YES
**Sparidae**							
*Archosargusrhomboidalis* (Linnaeus, 1758)	Sea Bream	S17	2,8	1	SJ13, SJ3		
*Calamusbajonado* (Bloch & Schneider, 1801)	Jolthead Porgy	S17	2	1			
*Calamuscalamus* (Valenciennes, 1830)	Saucereye Porgy	S17	2,4	1			
*Calamuspenna* (Valenciennes, 1830)	Sheepshead Porgy	S17	2,4	1			
*Calamuspennatula* Guichenot, 1868	Pluma Porgy	S17	2,4	1			YES
*Calamusproridens* Jordan & Gilbert, 1884	Littlehead Porgy			CMN			
*Diploduscaudimacula* (Poey, 1860)	Silver Porgy	S17	2,4,11	1	ST6		
**Sphyraenidae**							
*Sphyraenabarracuda* (Edwards, 1771)	Great Barracuda	S17	2,4,5,6,8	1			YES
*Sphyraenaborealis* DeKay, 1842	Sennet	S17	2	1	SJ13, SJ12, SJ21		
**Sphyrnidae**							
*Sphyrnalewini* (Griffith & Smith, 1834)	Scalloped Hammerhead		10	1			
*Sphyrnamokarran* (Rüppell, 1837)	Great Hammerhead		10				
**Spratelloididae**							
*Jenkinsialamprotaenia* (Gosse, 1851)	Dwarf Herring		2,6	1		1	YES
*Jenkinsiaparvula* Cervigón & Velazquez, 1978	Shortstriped Round Herring		2				
*Jenkinsiastolifera* (Jordan & Gilbert, 1884)	Shortband Herring		2				
**Squalidae**							
*Squaluscubensis* Howell Rivero, 1936	Cuban Dogfish			FlMNH			
**Sternoptychidae**							
*Sonodapaucilampa* Grey, 1960	Deepsea Hatchetfish			NMNH			
**Stomiidae**							
*Astronesthessimilus* Parr, 1927	Similar Snaggletooth			NMNH			
**Syngnathidae**							
*Amphelikturusdendriticus* (Barbour, 1905)	Pipehorse	S18			SJ12		
*Bryxdunckeri* (Metzelaar, 1919)	Pugnose Pipefish	S18	2	1	SJ13	1	YES
*Cosmocampusbrachycephalus* (Poey, 1868)	Crested Pipefish		2			1	
*Cosmocampuselucens* (Poey, 1868)	Shortfin Pipefish	S18	2,4	1	SJ19		
*Cosmocampusprofundus* (Herald, 1965)	Deepwater Pipefish		2				
*Halicampuscrinitus* (Jenyns, 1842)	Banded Pipefish	S18			SJ34, SJ13, SJ22		
*Hippocampuserectus* Perry, 1810	Lined Seahorse		11	1			YES
*Hippocampusreidi* Ginsburg, 1933	Longsnout Seahorse	S18	4	1	SJ19		YES
*Microphislineatus* (Kaup, 1856)	Opposum Pipefish	S18			O23		
*Pseudophallusmindii* (Meek & Hildebrand, 1923)	Freshwater Pipefish		11				
*Syngnathuscaribbaeus* Dawson, 1979	Caribbean Pipefish	S18	2		SJ21		
*Syngnathusdawsoni* (Herald, 1969)	Antillean Pipefish		2,4,11	1			
*Syngnathuspelagicus* Linnaeus, 1758	Sargassum Pipefish			ROM			
**Synodontidae**							
*Sauridabrasiliensis* Norman, 1935	Largescale Lizardfish		2				
*Sauridasuspicio* Breder, 1927	Doubtful Lizardfish	S18	2	1	SJ5, SJ13		YES
*Synodusfoetens* (Linnaeus, 1766)	Inshore Lizardfish	S18	2	1	SJ5, SJ13	1	YES
*Synodusintermedius* (Spix & Agassiz, 1829)	Sand Diver	S18	2,4	1		1	YES
*Synoduspoeyi* Jordan, 1887	Offshore Lizardfish		2				
*Synodussynodus* (Linnaeus, 1758)	Red Lizardfish	S18	2	1	SJ11, SJ21	1	
*Trachinocephalusmyops* (Forster, 1801)	Snakefish			CAS			
**Tetraodontidae**							
*Canthigasterrostrata* (Bloch, 1786)	Sharpnose Puffer	S18	2,4,5,8	1		1	
*Sphoeroidesspengleri* (Bloch, 1785)	Bandtail Puffer	S18	2,4	1		1	YES
*Sphoeroidestestudineus* (Linnaeus, 1758)	Checkered Puffer	S18	2,4,6	1	O15	1	
**Triakidae**							
*Musteluscanis* (Mitchill, 1815)	Smooth Dogfish			FlMNH			
**Triglidae**							
*Peristedionlongispatha* Goode & Bean, 1886	Widehead Armored Searobin			CAS			
**Tripterygiidae**							
*Enneanectesaltivelis* Rosenblatt, 1960	Lofty Triplefin	S18	2	1		1	
*Enneanectesatrorus* Rosenblatt, 1960	Blackedge Triplefin		2,11	1			
*Enneanectesboehlkei* Rosenblatt, 1960	Roughhead Triplefin	S18	2	1		-1	YES
*Enneanectesjordani* (Evermann & Marsh, 1899)	Mimic Triplefin	S18	2	1	SJ21		
*Enneanectesmatador* Victor, 2013	Matador Triplefin	S18		1			YES
**Xiphiidae**							
*Xiphiasgladius* Linnaeus, 1758	Swordfish	S18					

**Notes**: Image voucher – supplementary plate number is given; photographer name is imbedded in each image. Literature source – 1 DeAngelis et al. (2008); 2 Dennis (2000); 3 Dennis et al. (2004); 4 Friedlander et al. (2013); 5 Garcia-Sais (2005); 6 Loftus (2003); 7 Mantatrust.org pers. comm. to DRR; 8 Nelson and Appledorn (1985); 9 Quatrinni et al. (2017); 10 Recksiek et al. (2006), 11 Smith-Vaniz and Jelks (2014); 12 Rogers et al. (2010). Online source – 1 indicates that an aggregator source exists, with the source named whenever it represents the sole voucher: AMNH (American Museum of Natural History); NOAA (National Oceanographic and Atmospheric Administration); BOLD (Barcode of Life); FlMNH (Florida Museum of Natural History); MCZ (Museum of Comparative Zoology); NMNH (National Museum of Natural History); ANSP (Academy of Natural Sciences of Philadelphia); CAS (California Academy of Sciences); ROM (Royal Ontario Museum); KUBI (University of Kansas Biodiversity Institute); CMN (Canadian Museum of Nature). Uncommon – species seen at 3 or less named sites by CJE and AME (see Suppl material 3: File S2a, b (for site codes) and Suppl. material 4: File S3). Ichthyocide – species collected by this method as noted in Dennis (2000); parentheses indicate ichthyocide was the only collection method noted by Dennis (2000). Gobiidae – we follow Thacker (2009) in including Cerdale and Ptereleotris among the Gobiidae. *Hypoplectrus* – we follow Puebla et al. (2022) in treating *H.maculiferus* as a synonym of *H.aberrans*.

Dennis (2000) listed 401 species from 216 genera and 79 families from those two islands (Table 2). We found records of an additional 159 species, producing an increase of 39.7% in the number of species, 37.0% in the number of genera and 36.7% in the number of families known from there (Table 3). The additions include 34 species for which the only source is a voucher image, 50 species recorded in post-2000 publications, and 49 species recorded only by online sources of museum (and other) data (Table 3). Of the 561 in Table 2, 24.6% were uncommon. Although 30.1% were collected using rotenone, species accounts by Dennis (2000) mentioned no other collecting method for only 10.4% of that subgroup of species. The 561 include three non-natives to the area (*Oreochromisniloticus*, *Poeciliareticulata* and *Pteroisvolitans*), 11 freshwater/estuarine species (*Anguillarostratus*, *Dormitatormaculatus*, *Eleotrisperniger*, *Gobiomorusdormitor*, *Awaousbanana*, *Sicydiumplumieri*, *Sicydiumpunctatum*, *Dajausmonticola*, *Microphislineatus* and *Pseudophallusmindii* and 547 marine species native to the GC.

**Table 3.** Fishes from St. John-Thomas recorded by different sources.

Types of fish taxa recorded	Species	Genera	Families
**Total from all sources**	561	296	108
**From Literature sources** All	451	251	89
Dennis 2000 All	401	216	79
Sole source is Dennis 2000	164	126	55
Sources other than Dennis 2000	50	44	25
**From Online sources** All	453	253	97
Online sources only	50	46	42
**From Images** All	371	20	73
Images only	34	29	20
**Deep species** All sources	49	44	33
Recorded by Dennis 2000	19	18	13
**Uncommon shallow species**	138	104	45
**Ichthyocide Collection** All	173	99	45
Ichthyocide only	18	15	11
**mtDNA BARCODES**	**Species**	**Genera**	**Families**
St. John-Thomas	156	93	41
Sole record is from barcode data	1	1	1
Puerto Rico	90	50	25
St. John-Thomas but not Puerto Rico	113	61	24
Puerto Rico but not St. John-Thomas	47	18	8
St. Croix	1	1	1
British Virgin Islands	3	2	1
All sites combined	207	112	49

**Notes**: Data sources (literature, online sources, images) are listed in Table 2. Deep species are those exclusively or typically found below 40 m depth. Uncommon shallow species are those found at 1–3 sites by CJE, AME, LR, and third-party photographers as indicated in Table 2. Ichthyocide collection: recorded as being collected with rotenone by a source cited by Dennis (2000). Ichthyocide only: the only collection method listed for a species from St. John-Thomas by Dennis (2000). DNA barcodes: (see Suppl. material 7: File S6). The single DNA Barcoded species collected at St. Croix (see Suppl. material 7: File S6) is not known from St. John-Thomas. The St. John-Thomas species count includes four identified only to genus. DNA barcode data for *Pteroisvolitans* are not included in this table.

### ﻿Comparative taxonomic composition of the USVI fish faunas (Table 4, Suppl. material 5: File S4)

The species richness of the USVI marine fauna (i.e., the combined St. John-Thomas plus St. Croix faunas) was 15–20% greater than that of either of the two insular faunas (Table 4). Those two faunas had slightly higher relative rates of richness of genera and families than of species. The larger size of the USVI fauna of species derives from ~ 1/5 of species in each insular fauna not being present in the other, with lower proportions of genera and families also being recorded only at one of the two islands. Relative faunal richness at all three taxonomic levels and the relative abundance of taxa present at only one island were ~ 5% higher at St. Croix than St. John-Thomas. In both island faunas the relative representation of species, genera, and families in the entire USVI fauna was substantially greater among shallow species than deep species. The deep fauna was much smaller than the shallow fauna at each island and there was much less overlap in occurrence of species, genera, and families between the two insular deep faunas than between their shallow faunas (Table 4).

**Table 4.** Taxonomic comparisons of St. John-Thomas and St. Croix marine fish faunas.

Site	Species	Genera	Families
**Both US Virgin Islands**			
Entire fauna (n)	679	345	122
Shallow fishes (n)	590	279	90
Deep fishes (n)	89	77	54
**St. John-Thomas**			
Entire Fauna (n)	547	283	105
Percent of USVI fauna	80.6	82.0	86.0
Percent only at St. John-Thomas	19.3	15.5	10.5
Shallow fishes (n)	497	245	86
Percent of USVI shallow fauna	84.2	86.6	94.5
Percent only at St. John-Thomas	13.0	7.4	1.9
Deep fishes (n)	50	44	34
Percent of USVI deep fauna	56.2	57.1	63.0
Percent only at St. John-Thomas	70.0	50.0	26.5
**St. Croix**			
Entire fauna (n)	573	301	112
Percent of USVI fauna	84.5	87.2	91.8
Percent only at St. Croix	23.4	20.4	15.5
Shallow fishes (n)	519	256	88
Percent of USVI fauna	88.0	91.8	97.8
Percent only at St. Croix	18.3	13.1	2.7
Deep fishes (n)	54	50	39
Percent of USVI deep fauna	61.4	64.9	62.2
Percent only St. Croix	72.2	60.0	41.0

**Notes**: USVI fauna = combined fauna of St. John-Thomas and St. Croix, with exotic and primarily freshwater species excluded. Some genera and families have a deep member in one site but not the other, which affects USVI totals for deep and shallow genera and families. Shallow fishes: species exclusively or commonly found shallower than 40 m. Deep fishes: species exclusively or largely found deeper than 40 m (see methods for further details).

### ﻿Ecotypic structure of the USVI reef-fish faunas vs. the region (Table 5, Suppl. material 6: File S5)

We compared the ecotypic structure of the St. John-Thomas and St. Croix faunas of reef-associated fishes with that of the GC fauna (see Robertson and Tornabene 2021). Both St. Croix and St. John-Thomas have faunas that are almost half the size of the total regional fauna, with the listed St. John-Thomas fauna being ~ 5% smaller than that of St. Croix (Table 5). Compared to the GC fauna both islands have slightly higher percentages of pelagic species, distinctly higher percentages of demersal species, and correspondingly lower percentages of benthic, cryptobenthic, small cryptobenthic, and CCRF species. These differences for non-pelagic types apply to each entire USVI fauna, and to both shallow- and deep-reef subgroups of those faunas. Both USVI sites also have markedly lower relative abundances (~ 1/3) of deep-reef species than the regional fauna. The relative abundances of different ecotypes are remarkably similar at both islands, except for the presence of a few deep cryptobenthic and CCRF species detected only at St. John-Thomas.

**Table 5.** Abundance of ecotypes of reef-associated bony fishes in the Greater Caribbean and the USVI.

	**Region**	**St. John-Thomas**	**St. Croix**
**All species (*n*)**	992	470	493
**Pelagic species % of fauna**	8.0	**10.4**	**10.3**
**Non-pelagic species % of fauna**	92.0	89.6	89.7
Demersal species %	34.6	**46.3**	**45.0**
Benthic species %	**65.4**	53.7	55.0
Cryptobenthic species %	**64.6**	53.0	54.3
Small cryptobenthic species %	**42.6**	31.6	32.5
CCRF species %	**45.9**	36.3	35.7
**SHALLOW NON-PELAGIC SPECIES (n)**	772	400	424
**Percent of fauna**	84.6	**95.0**	**95.9**
Demersal species %	34.9	**45.3**	**44.0**
Benthic species %	**65.1**	54.7	56.0
Cryptobenthic species %	**64.0**	54.3	55.2
Small cryptobenthic species %	**42.5**	33.3	34.0
CCRF species %	**46.0**	37.5	37.3
**DEEP NON-PELAGIC SPECIES (n)**	141	21	18
**Percent of fauna**	**15.4**	5.0	4.2
Demersal species %	33.3	**66.7**	**66.7**
Benthic species %	**66.7**	33.3	33.3
Cryptobenthic species %	**66.7**	33.3	33.3
Small cryptobenthic species %	**43.3**	4.8	0
CCRF species %	**45.4**	19.0	0

**Notes**: Data for the region pattern are from Robertson and Tornabene (2021), for St. Croix are from Robertson et al. (2022), and for St. John-Thomas are in File S5. Bold percentages indicate whether the value(s) for either the region or the USVI islands were > 5% higher than the value(s) for the other group in each case.

### ﻿Zoogeographic structure of the USVI faunas (Table 6)

The zoogeographic structures of the faunas of the two USVI sites and nearby Sint Eustatius are quite similar (Table 6). Species that are endemic to the Greater Caribbean and, in a few cases, surrounding areas are the largest group in all three faunas, with West Atlantic species also found in Brazil being the second largest by a small margin in each case. The two smallest groups in each case are Trans-Atlantic and Atlantic & Indo-Pacific. The ranks of those four groups are the same in all three faunas, a measure of their strong similarities.

**Table 6.** Zoogeographic composition of the USVI and Sint Eustatius faunas. Percentage of species in each category. Non-native species are not included.

Site (*n*)	Northwest Atlantic	Western Atlantic	Trans-Atlantic	Atlantic & Indo-Pacific
St. Croix (534)	41.6	33.9	13.9	10.6
St. John-Thomas (558)	39.5	36.5	14.0	10.0
Sint Eustatius (406)	41.1	33.3	15.3	10.3

**Notes**: St. Croix data are from Smith-Vaniz and Jelks 2014. Sint Eustatius values are from Robertson et al. (2020). St. John-Thomas values are from the present study. Northwestern Atlantic = Greater Caribbean, with or without range extensions to the north of that region. Western Atlantic = Northwestern Atlantic + Brazil. Trans-Atlantic = anywhere in the western Atlantic + any of the islands of the central Atlantic and/or the Eastern Atlantic. Atlantic & Indo-Pacific = Anywhere in the Western Atlantic + anywhere in the Indo-Pacific.

### ﻿mtDNA-Barcode Coverage (Tables 2, 3; Suppl. material 7: File S6)

Table 2 indicates which members of the St. John-Thomas fauna have mtDNA-barcode sequences on the BOLD database derived from specimens collected at that site. Table 3 presents a summary of taxa that have sequences obtained from St. John-Thomas, Puerto Rico, the British Virgin Islands and St. Croix, singly and in combination. File S6 provides technical information about those barcode data for the various species. We obtained local DNA-barcodes for 156 fish species in 156 BINs from St. John-Thomas, with one additional from St. Croix, and three additional species from around the British Virgin Islands (total 160 species). Of these, two are only from GenBank records harvested by BOLD, and 10 are added from specimens collected in offshore larval plankton tows described in Lamkin et al. (2009). We obtained 91 species records (including one non-native, *Pteroisvolitans*) for Puerto Rico, 44 of them shared with the Virgin Islands. Of the 91, 27 are added from Harms-Tuohy et al. (2016), 14 from GenBank records harvested by BOLD, and seven from other sources, including the University of Kansas (UKFBJ), Smithsonian (Bermingham/Lessios; BSMUA & BSOPA), the Guy Harvey Research Institute (Hanner et al. 2011; EBFSF), and the Museum and Art Gallery of the Northern Territory (GOBY) in Australia.

The available DNA-barcode sequence records from specimens collected at St. John-Thomas represent coverage of 27.8% of the species, 31.4% of the genera and 38% of the families of fishes known from that site. Barcode records represent the sole source of information on the presence of one species known from those islands and are also available for another four species currently identifiable only to genus. Distinctly fewer species have been barcoded from fish taken at Puerto Rico, and there are almost no such data available from either St. Croix or the British Virgin Islands. Barcode records from Puerto Rico and the British Virgin Islands exist for 52 species occurring in St. John-Thomas but not sequenced from there, bringing the total PRP DNA-barcoded species to 36.5% of St. John-Thomas fauna. All but seven of the 200 barcoded species are reef-associated bony fishes. The vast majority (98.5%) of barcoded species are shallow forms. Deep-living species are especially under-represented among the barcoded forms: only three of 51 such species have barcode data (File S6).

## ﻿Discussion

### ﻿St. Croix

The species records we have added increased the size of that island’s fauna by 7.5%. Almost a third of the additions arise from voucher photographs of shallow-reef species photographed by CJE and AME (and provided by Mantatrust.org). Those include several not accepted by Smith-Vaniz and Jelks (2014) due to inadequate information available at that time. Cryptobenthic fishes, which, by definition, are generally difficult to observe, are a major component of Greater Caribbean reef-fish faunas, including that of St. Croix. Such species comprised all but one of those added by CJE and AME. The exception, *Kyphosuscinerascens*, may have been misidentified previously, since the taxonomic status and global distributions of members of the genus were only comprehensively reviewed by Knudsen and Clements (2016), after Smith-Vaniz and Jelks (2014) published their checklist. Almost half the additions were deep-living species, one third of which were recorded only by submersible or ROV, with the remainder coming from online and literature records.

The process of obtaining location records is an ongoing one for online aggregators, which have vastly increased the amounts of data they host during the last half decade. Although the aggregators offer such information, and are involved in collaborative data sharing, such sharing is sufficiently incomplete that it is necessary to examine records from multiple aggregators to obtain a comprehensive picture of all the data available for any particular site. Even “old” data becomes newly available on the aggregators from time to time and needs to be included in faunal inventories of well-studied sites. The increase in faunal size, although not large in percentage terms, demonstrates the utility of citizen-science efforts to provide photographic vouchers, of reviews of submersible and ROV studies of deep-living fishes, and of periodic evaluations of information available online from aggregators.

### ﻿St. John-Thomas

Although the 401 species list for this site extracted from Dennis (2000) was substantial (74% the size of Smith-Vaniz and Jelks (2014) count for St. Croix), our use of the same methods as those that produced an increase in the St. Croix fauna produced a much larger increase in the St. John-Thomas fauna: 40% vs. 7.5% for St. Croix. Dennis (2000) was the sole source for 29% of species recorded in our expanded list of the St. John-Thomas fauna. Records from additional sources brought the size of the St. John-Thomas fauna to within 5% of the size of the St. Croix fauna. Citizen-scientists’ photographic records accounted for 22% of the new additions and data only available from online databases for 33%, while other literature sources provided the sole records for 32% of the additional species. Multiple types of sources accounted for the remaining 13% of new records.

### ﻿The size, and taxonomic- and ecotypic structure of the two USVI marine faunas

Both insular marine faunas are over 80% the size of the combined USVI fauna in terms of species richness. Species found at only one of the two islands represent ~ 20% of each fauna. For shallow species the size of each insular fauna is 85–90% that of the combined fauna, with correspondingly lower rates of occurrence at only one island. Two factors may contribute to these differences between the island faunas: variation in ecological conditions between the islands and inadequate sampling. The possibility of differing ecological conditions seems small as both islands have the same range of large-scale habitat types, although those vary in abundance between the islands. The shelf area of St. John-Thomas is close to 10 times the size of the St. Croix shelf, yet the former has the smaller known fauna. At both islands the great majority of sampling has occurred in quite shallow water, often very close to shore in the case of St John-Thomas. Shelf habitats likely are under-sampled at both islands, strongly so at St. John-Thomas, where there are large areas of habitat between 40–60 m depth some distance from the islands on both the northern and southern parts of the PRP. At St. Croix most shallow sampling has occurred in and near the Buck Island Reef National Monument, rather than spread around different parts of the platform and different sides of the island. Hence both insular faunas likely are larger than indicated here, particularly in the case of St. John-Thomas.

Review of the two USVI marine species lists show that species not shared between the two islands are distributed through many genera and families (Suppl. material 5: File S4; Table 4). None are endemic to either USVI island and single-island endemics are rare amongst the Greater Caribbean fauna and limited to highly isolated islands such as Cayman. Most species in that region have geographic ranges much larger than the USVIs. The larger size of the St. Croix fauna, particularly of cryptobenthic species can be attributed to a greater effort to find such species. This was done using rotenone during two intensive sampling campaigns that occurred ~ 40 y after rotenone sampling at St. John-Thomas, plus some subsequent minor efforts in the shallow part of a Buck Island Reef National Monument that, in its entirety constitutes ~ 1/3 of the St. Croix insular platform: 46% (262) of the native marine species known from St. Croix are shallow species collected using rotenone (Smith-Vaniz and Jelks 2014), vs. 31.7% (173) of such species from St. John-Thomas. Later sampling by Pittman et al. (2008) at the same small, shallow St. Croix site as studied by Smith-Vaniz et al. (2006) added 10.9% more species to the tally of the first two series of collections. Smith-Vaniz and Jelks (2014) produced a list of 41 species from 22 families that, at that time, were known from St. John-Thomas but not St. Croix. Since then, five of the 35 shallow species on that table have been added to the St. Croix fauna (Table 1 here), together with two others that were listed as unconfirmed by those authors. Photographic sampling of shallow reef fishes at St. John-Thomas by CJE, AME and other citizen scientists, by itself increased the size of the fauna registered by Dennis (2000) by 8.5%. Finally, the species composition of local reef-fish faunas can change substantially through time at intensively sampled sites, for varying reasons (e.g., see changes registered by Starck et al. 2017 over a 50y period), highlighting the utility of temporally dispersed sampling. With further sampling many shallow species currently known from only one of the USVI should be expected to be found at the other, in which case the shallow faunas of each island would be 10–15% larger than the current figures.

The deep-species fauna represents only 13.1% of the entire (shallow plus deep) USVI fauna and deep species exhibit much lower rates of faunal overlap between the two islands than occurs among shallow species. The two islands have experienced low rates of exploration of deep habitats, particularly deep reefs, by submersibles and ROVs, which were limited to observational studies. The few ROV (Quattrini et al. 2017) and submersible dives (Nelson and Appeldoorn 1985; Garcia-Sais 2005) were the sole source of only 11.1% and 28% of records of deep fishes at St. Croix and St. John-Thomas, respectively. The edges of the insular platforms of the two USVIs are < 50 km apart and the suite of deep species involved have ranges much larger than the area occupied by the USVI. Low levels of sampling can account for the small size of both USVI deep faunas, particularly the deep-reef component, and to the low level of overlap between the deep faunas of the two islands.

At both USVI sites the deep-reef species represent only 4.2–5% of the entire local reef-fish fauna, i.e., ~ 1/3 of the percentage for the GC regional fauna (Robertson et al. 2022). In contrast, when intensive submersible collecting and observations have been aimed specifically at assessing the diversity of deep-reef fish faunas, such as has occurred at other Caribbean islands (Curacao, Roatan and Sint Eustatius), the inventory of deep-reef species at individual islands has increased ~ 9 fold, with such species representing 16% of the entire (shallow plus deep) reef-fish fauna at the most intensively sampled island (Robertson et al. 2022), i.e., more than three times the level at each USVI. Similar sampling at both USVI undoubtedly will increase the absolute and relative sizes of their deep-reef faunas. Smith-Vaniz and Jelks (2014) concluded that there was no indication at the time of their study that the St. Croix fauna had reached asymptotic size. The additions reported here and patterns of variation in faunal composition between the two islands support that view for St. John-Thomas as well as St. Croix.

Reef-associated bony fishes comprised 84% and 91%, respectively, of the faunas of St. John-Thomas and St. Croix, and the St. John-Thomas reef-fish fauna was 94.3% the size of the equivalent fauna of St. Croix. The ecotypic structure of those two USVI reef-fish faunas was very similar, with both differing from the broad structure of the GC regional fauna by having larger proportions of pelagic and demersal species that are readily visible to observers and correspondingly smaller proportions of cryptic species. Similarities in the zoogeographic structures and sizes of the two USVI faunas support the view that both can be considered to be sampled with a similar level of efficiency, at least in terms of their shallow faunas.

### ﻿mtDNA-barcode coverage

In terms of the availability of DNA-barcodes for marine fishes, the Greater Caribbean currently is the most well-sampled large marine biogeographic region in the tropics, with ~ 90% of the shore-fishes barcoded and up to 95% of the shallow reef-associated species (Victor et al. 2015). However, several specific locations account for the vast majority of sequences. Those include Florida, Yucatan (Mexico), Belize, Panama, and Curacao; with species lists published for Yucatan by Valdez-Moreno et al. (2010) and lists for additional locations in Weigt et al. (2012). The Puerto Rican Plateau has been only lightly sampled, with information derived mostly from older collections by author BV at St. John-Thomas and Puerto Rico, and from a set of lionfish stomach contents from La Parguera in Puerto Rico sequenced by Harms-Tuohy et al. (2016). The latter identified 39 species from 16 families. A few additional sequences come from open-ocean sampling for larvae around the USVI, by Lamkin et al. (2009). The older collections from St. Thomas and Puerto Rico were collected by BV for recruitment and otolith studies as well as some taxonomic reviews (e.g., the genera *Coryphopterus* and *Emblemariopsis*). The recent additions of 19 species from St. John were collected by CJE and AME mainly for DNA confirmation of the species identification of diagnostic underwater photographs that serve as vouchers here, mostly of cryptobenthic fishes. No collections at St. John-Thomas or elsewhere on the PRP that provided DNA barcodes were expressly made for assembling an inventory of fish species- hence the absence of some of the most abundant and widespread shallow reef fishes in the barcode list presented here (e.g., the Bluehead Wrasse, *Thalassomabifasciatum*).

We cannot directly compare barcode coverage of fishes at St. John-Thomas with that at other intensively barcoded locations noted above because neither the number of barcoded species nor the local species inventory have been comparably evaluated at any of those sites. The results of the present assessment of DNA-barcode coverage for the USVI and the remainder of the PRP highlight the usefulness of the DNA-barcode database for ancillary projects. Accumulating sequences for unrelated purposes, such as taxonomic reviews, stomach-content studies, larval or e-DNA surveys (environmental DNA, where water is sampled for dissolved DNA sequences), augments the general DNA-barcode coverage for specific biogeographic regions and helps confirm species identifications for faunal surveys.

## ﻿Permits

Collections from St. John in 2021 were made under National Park Service Collecting Permit VIIS-2021-SCI-0006: We especially thank Thomas Kelley (Virgin Islands NP/Virgin Islands Coral Reef NM) for facilitating that work.

## Supplementary materials

Supplementary material 1 Plates S1–S18 Data type images (jpg. images in ZIP arhiv) Explanation note Fishes of St. Croix (Plate S1), fishes of St. John-Thomas (Plates S2–S18). https://binary.pensoft.net/file/691027This dataset is made available under the Open Database License (http://opendatacommons.org/licenses/odbl/1.0/). The Open Database License (ODbL) is a license agreement intended to allow users to freely share, modify, and use this Dataset while maintaining this same freedom for others, provided that the original source and author(s) are credited.D. Ross Robertson, Carlos J. Estapé, Allison M. Estapé, Lee Richter, Ernesto Peña, Benjamin Victor

Supplementary material 2 File S1 Data type image (jpg file) Explanation note Bathymetry of the US Virgin Islands. https://binary.pensoft.net/file/691028This dataset is made available under the Open Database License (http://opendatacommons.org/licenses/odbl/1.0/). The Open Database License (ODbL) is a license agreement intended to allow users to freely share, modify, and use this Dataset while maintaining this same freedom for others, provided that the original source and author(s) are credited.D. Ross Robertson, Carlos J. Estapé, Allison M. Estapé, Lee Richter, Ernesto Peña, Benjamin Victor

Supplementary material 3 File S2 Data type GPS data (excel file) Explanation note File S2A: Georeferencing coordinates and site codes for dive sites of authors Carlos and Allison Estapé at St John, St Thomas and St. Croix during 2021. File S2B: Georeferencing coordinates and site codes for dive sites used by non-author photographers at St John-Thomas. File S2C: Names and emails of third party Citizen Scientists who provided voucher images of various St John-Thomas fishes. https://binary.pensoft.net/file/691029This dataset is made available under the Open Database License (http://opendatacommons.org/licenses/odbl/1.0/). The Open Database License (ODbL) is a license agreement intended to allow users to freely share, modify, and use this Dataset while maintaining this same freedom for others, provided that the original source and author(s) are credited.D. Ross Robertson, Carlos J. Estapé, Allison M. Estapé, Lee Richter, Ernesto Peña, Benjamin Victor

Supplementary material 4 File S3 Data type GPS data (kmz. file) Explanation note KMZ file of USVI dive sites. https://binary.pensoft.net/file/691030This dataset is made available under the Open Database License (http://opendatacommons.org/licenses/odbl/1.0/). The Open Database License (ODbL) is a license agreement intended to allow users to freely share, modify, and use this Dataset while maintaining this same freedom for others, provided that the original source and author(s) are credited.D. Ross Robertson, Carlos J. Estapé, Allison M. Estapé, Lee Richter, Ernesto Peña, Benjamin Victor

Supplementary material 5 File S4 Data type occurrences (excel file) Explanation note File S4. Native marine fish faunas of St. John-Thomas and St. Croix. https://binary.pensoft.net/file/691031This dataset is made available under the Open Database License (http://opendatacommons.org/licenses/odbl/1.0/). The Open Database License (ODbL) is a license agreement intended to allow users to freely share, modify, and use this Dataset while maintaining this same freedom for others, provided that the original source and author(s) are credited.D. Ross Robertson, Carlos J. Estapé, Allison M. Estapé, Lee Richter, Ernesto Peña, Benjamin Victor

Supplementary material 6 File S5 Data type occurenses (excel file) Explanation note Ecological Characteristics of Reef-Associated Bony Fishes from St John-Thomas. See Methods of paper for details. https://binary.pensoft.net/file/691032This dataset is made available under the Open Database License (http://opendatacommons.org/licenses/odbl/1.0/). The Open Database License (ODbL) is a license agreement intended to allow users to freely share, modify, and use this Dataset while maintaining this same freedom for others, provided that the original source and author(s) are credited.D. Ross Robertson, Carlos J. Estapé, Allison M. Estapé, Lee Richter, Ernesto Peña, Benjamin Victor

Supplementary material 7 File S6 Data type genomic (excel file) Explanation note File S6: mtDNA-Barcode information for fishes from islands on the Puerto Rico Platform (St John-Thomas, Puerto Rico and the British Virgin Islands) and St. Croix. For coding of differently colored highlighting see bottom of table. For explanations of "Reef Associated" and "Deep" see main text. https://binary.pensoft.net/file/691033This dataset is made available under the Open Database License (http://opendatacommons.org/licenses/odbl/1.0/). The Open Database License (ODbL) is a license agreement intended to allow users to freely share, modify, and use this Dataset while maintaining this same freedom for others, provided that the original source and author(s) are credited.D. Ross Robertson, Carlos J. Estapé, Allison M. Estapé, Lee Richter, Ernesto Peña, Benjamin Victor

## References

[B1] Baldwin CC Tornabene L Robertson DR (2018) Below the mesophotic. Scientific Reports 8: e4920. 10.1038/s41598-018-23067-1 PMC5861108 29559694

[B2] Brandl SJ Goatley CHR Bellwood DR Tornabene L (2018) The hidden half: Ecology and evolution of cryptobenthic fishes on coral reefs. Biological Reviews of the Cambridge Philosophical Society 93(4): 1846–1873. 10.1111/brv.12423 29736999

[B3] Bunckley-Williams L Williams EH (2004) New Locality, Depth, and Size Records and Species Character Modifications of Some Caribbean Deep-Reef/Shallow Slope Fishes and a New Host and Locality Record for the Chimaera Cestodarian. Caribbean Journal of Science 40: 88–119.

[B4] Clavijo IE Yntema JA Ogden JC (1980) An annotated list of the fishes of St. Croix, U.S. Virgin Islands. West Indies Lab, Special Publication, 2^nd^ edn. West Indies Laboratory, Christiansted, 49 pp. 10.5281/zenodo.5510695

[B5] DeAngelis BM McCandless CT Kohler NE Recksiek CW Skomal GB (2008) First characterization of shark nursery habitat in the United States Virgin Islands: Evidence of habitat partitioning by two shark species. Marine Ecology Progress Series 358: 257–271. 10.3354/meps07308

[B6] Dennis GD (2000) Annotated checklist of shallow-water marine fishes from the Puerto Rico Plateau including Puerto Rico, Culebra, Vieques, St. Thomas, St. John, Tortola, Virgin Gorda and Anegada. Florida Caribbean Science Center, Gainesville, [244 + 26] 286 pp. 10.5281/zenodo.5770763

[B7] Dennis GD Hensley D Colin PL Kimmel JJ (2004) New Records of Marine Fishes from the Puerto Rican Plateau. Caribbean Journal of Science 40: 70–87. 10.5281/zenodo.5512853

[B8] Friedlander AM Jeffrey CFG Hile SD Pittman SJ Monaco ME Caldow C [Eds] (2013) Coral reef ecosystems of St. John, U.S. Virgin Islands: Spatial and temporal patterns in fish and benthic communities (2001–2009). NOAA Technical Memorandum 152. Silver Spring, MD, 150 pp. https://repository.library.noaa.gov/view/noaa/789

[B9] García-Sais JR (2005) Inventory and Atlas of Corals and Coral Reefs, with Emphasis on Deep-Water Coral Reefs from the U. S. Caribbean EEZ Final Report; Caribbean Fishery Management Council San Juan, Puerto Rico, 214 pp. http://sedarweb.org/docs/wsupp/S26_RD_02_deep_reefs_report_2005.pdf

[B10] García-Sais JR Williams SM Sabater-Clavell J Esteves R Carlo M (2014) Mesophotic benthic habitats and associated reef communities at Lang Bank, St. Croix, USVI. Final Report; Caribbean Fishery Management Council San Juan, Puerto Rico, 124 pp.

[B11] Hanner R Floyd R Bernard A Collette BB Shivji M (2011) DNA barcoding of billfishes. Mitochondrial DNA 22(sup1, S1): 27–36. 10.3109/19401736.2011.596833 21980985

[B12] Harms-Tuohy CA Schizas NV Appeldoorn RS (2016) Use of DNA metabarcoding for stomach content analysis in the invasive lionfish *Pteroisvolitans* in Puerto Rico. Marine Ecology Progress Series 558: 181–191. 10.3354/meps11738

[B13] Knudsen SW Clements KC (2016) World-wide species distributions in the family Kyphosidae (Teleostei: Perciformes). Molecular Phylogenetics and Evolution 101: 252–266. 10.1016/j.ympev.2016.04.037 27143240

[B14] Lamkin JT Gerard TL Malca E Shiroza A Muhling BA Davis N Fuenmayor F Whitecraft S Johns L Smith R Melo N Rawson G Idrisi N Smith T Brown K (2009) USVI larval reef fish supply study: 2007-08 report. Coral Reef Conservation Program (U.S.). U.S. Dept. of Commerce, National Oceanic and Atmospheric Administration, Southeast Fisheries Science Center, Miami, 43 pp. https://repository.library.noaa.gov/view/noaa/548

[B15] Loftus WF (2003) Inventory of fishes in inland fresh and brackish-water habitats of Virgin Island National Park. Final Report, U.S. Inventory and Monitoring Program 3207–24F29 VIIS-1102, 52 pp. https://irma.nps.gov/DataStore/Reference/Profile/2175778

[B16] Nelson WR Appeldoorn RS (1985) Cruise Report R/V Seward Johnson. A Submersible Survey of the Continental Slope of Puerto Rico and the U.S. Virgin Islands, 1–23 October 1985, 76 pp. https://link.springer.com/chapter/10.1007/978-3-319-92735-0_7

[B17] Pittman SJ Hile SD Jeffrey CFG Caldow C Kendall MS Monaco ME Hillis-Starr Z (2008) Fish assemblages and benthic habitats of Buck Island Reef National Monument (St. Croix, U.S. Virgin Islands) and the surrounding seascape: A characterization of spatial and temporal patterns. NOAA Technical Memorandum NOS NCCOS 71. Silver Spring, MD, 96 pp. https://coastalscience.noaa.gov/data_reports/fish-assemblages-and-benthic-habitats-of-buck-island-reef-national-monument-st-croix-u-s-virgin-islands-and-the-surrounding-seascape-a-characterization-of-spatial-and-temporal-patterns-2/

[B18] Puebla O Coulmance F Estape CJ Morgan Estape A Robertson DR (2022) A review of 263 years of taxonomic research on *Hypoplectrus* (Perciformes: Serranidae), with a redescription of *Hypoplectrusaffinis* (Poey, 1861). Zootaxa 5093(2): 101–141. 10.11646/zootaxa.5093.2.1 35390813

[B19] Quattrini AM Demopoulos AWJ Singer R Roa-Varon A Chaytor JD (2017) Demersal fish assemblages on seamounts and other rugged features in the northeastern Caribbean. Deep-sea Research. Part I, Oceanographic Research Papers 123: 90–104. 10.1016/j.dsr.2017.03.009

[B20] Recksiek C Wetherbee BM DeAngelis B (2006) Assessment of the Status of Shark Populations in the USVI. Final Report. University of Rhode Island, Kingston, 22 pp. https://www.ncei.noaa.gov/data/oceans/coris/library/NOAA/CRCP/project/1413/NA04NMF4630343_FinalReport_shark_usvi.pdf

[B21] Robertson DR Tornabene L (2021) Reef-associated Bony Fishes of the Greater Caribbean: a Checklist (Version 4) [Data set]. Zenodo. 10.5281/zenodo.5592149

[B22] Robertson DR Estapé CJ Estapé AM Peña E Tornabene L Baldwin CC (2020) The marine fishes of St Eustatius Island, northeastern Caribbean: an annotated, photographic catalog. Zookeys 1007: 145–180. 10.3897/zookeys.1007.58515 33505184 PMC7788074

[B23] Robertson DR Tornabene L Lardizabal CC Baldwin CC (2022) Submersibles greatly enhance research on the diversity of deep-reef fishes in the Greater Caribbean. Frontiers in Marine Science 8: e800250. 10.3389/fmars.2021.800250

[B24] Rogers CS Pietsch TW Randall JE Arnold RJ (2010) The Sargassum Frog-fish (*Histriohistrio* Linnaeus) Observed in Mangroves in St. John, U.S. Virgin Islands. Coral Reefs 29(3): 577. 10.1007/s00338-010-0636-z

[B25] Rohmann SO Hayes JJ Newhall RC Monaco ME Grigg RW (2005) The area of potential shallow-water tropical and subtropical coral ecosystems in the United States. Coral Reefs 24(3): 370–383. 10.1007/s00338-005-0014-4

[B26] Smith-Vaniz W Jelks HL (2014) Marine and inland fishes of St. Croix, U. S. Virgin Islands: an annotated checklist. Zootaxa 3803: 1–120. 10.11646/zootaxa.3803.1.1 24871150

[B27] Smith-Vaniz W Jelks HL Rocha LA (2006) Relevance of cryptic fishes in biodiversity assessments: A case study at Buck Island Reef National Monument, St. Croix. Bulletin of Marine Science 79: 17–48. https://www.ingentaconnect.com/content/umrsmas/bullmar/2006/00000079/00000001/art00002

[B28] Starck WA Estapé CJ Morgan Estapé A (2017) The fishes of Alligator Reef and environs in the Florida Keys: A half-century update. Journal of the Ocean Science Foundation 27: 74–117. 10.5281/ZENODO.851651

[B29] Thacker CE (2009) Phylogeny of Gobioidei and placement within Acanthomorpha, with a new classification and investigation of diversification and character evolution. Copeia 2009(1): 93–104. 10.1643/CI-08-004

[B30] Valdez-Moreno M Vásquez-Yeomans L Elías-Gutiérrez M Ivanova NV Hebert PDN (2010) Using DNA barcodes to connect adults and early life stages of marine fishes from the Yucatan Peninsula, Mexico: Potential in fisheries management. Marine and Freshwater Research 61(6): 655–671. 10.1071/MF09222

[B31] Victor BC Valdez-Moreno M Vasquez-Yeomans L (2015) Status of DNA Barcoding Coverage for the Tropical Western Atlantic Shore-fishes and Reef Fishes. DNA Barcodes 3(1): 89–93. 10.1515/dna-2015-0011

[B32] Ward RD Hanner R Hebert PDN (2009) The campaign to DNA barcode all fishes, FISH-BOL. Journal of Fish Biology 74(2): 329–356. 10.1111/j.1095-8649.2008.02080.x 20735564

[B33] Weigt LA Baldwin CC Driskell A Smith DG Ormos A Reyler EA (2012) Using DNA Barcoding to Assess Caribbean Reef Fish Biodiversity: Expanding Taxonomic and Geographic Coverage. PLoS ONE 7(7): e41059. 10.1371/journal.pone.0041059 PMC3398874 22815912

