# ﻿Addendum and Corrigendum: Robertson DR, Estapé CJ, Estapé AM, Richter L, Peña E, Victor B (2022) An updated, illustrated inventory of the marine fishes of the US Virgin Islands. Zookeys 1103: 79–122. doi: 10.3897/zookeys.1103.83795

**DOI:** 10.3897/zookeys.1112.87591

**Published:** 2022-07-14

**Authors:** David Ross Robertson, Carlos J. Estapé, Allison M. Estapé, Lee Richter, Ernesto Peña, Benjamin Victor

**Affiliations:** 1 Smithsonian Tropical Research Institute, Balboa, Panam Smithsonian Tropical Research Institute Balboa Panama; 2 197 Gulfview Drive, Islamorada, Florida, 33036, USA Unaffiliated Islamorada United States of America; 3 National Park Service, 1300 Cruz Bay Creek, St. John, 00830, Virgin Islands, USA National Park Service St. John Virgin Islands (USA); 4 Ocean Science Foundation, 4051 Glenwood, Irvine, CA 92604, USA Ocean Science Foundation Irvine United States of America; 5 Guy Harvey Research Institute, Nova Southeastern University, 8000 North Ocean Drive, Dania Beach, FL 33004, USA Nova Southeastern University Dania Beach United States of America

**Keywords:** Biodiversity, Caribbean, identification, reef fishes

## Abstract

Review of the image plates shows that an image of *Rypticussubbifrenatus* was incorrectly identified as that of its similarly colored congener *R.carpenter*. Hence the latter was deleted from the St. John-Thomas inventory. In addition, an image of the blenniid fish *Hypsoblenniusexstochilus* was obtained from St. Thomas, and it is now added to that inventory. These two changes did not substantially affect data on the ecological structure of the St. John-Thomas fauna.

## ﻿Introduction

Prompt publication of corrections to faunal inventories helps ensure the accuracy of biogeographical data. Here we correct the erroneous occurrence of a misidentified serranid fish in the St. John-Thomas inventory. In addition, a newly available photograph of a blenniid taken at St. Thomas provides a voucher for its addition to that inventory.

**Figure 1.**
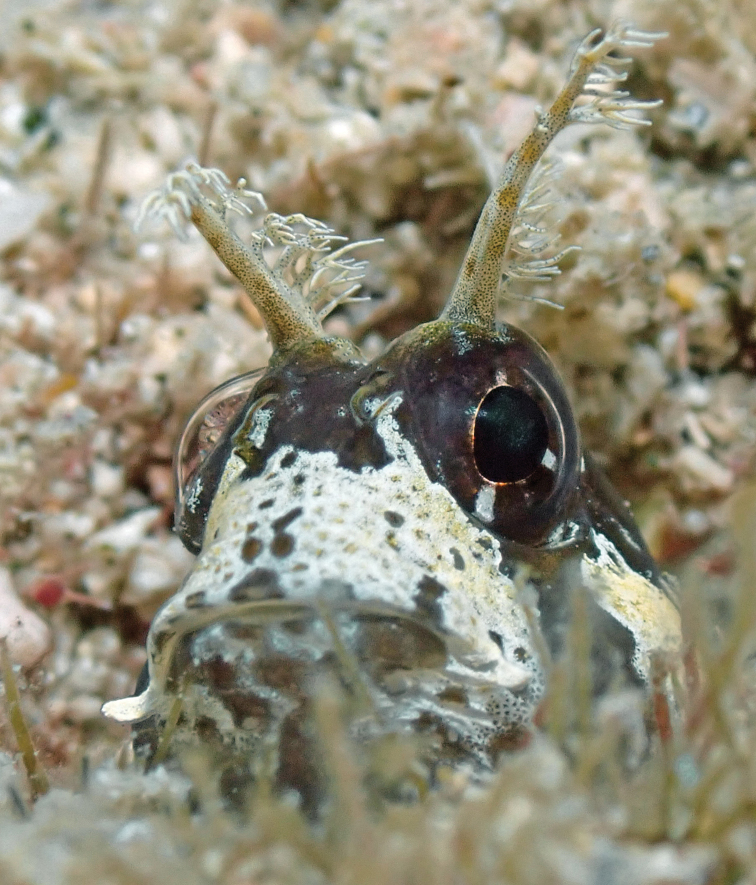
*Hypsoblenniusexstochilus*. Photo: Natasha Bestrom (natasha.bestrom@uvi.edu) St Thomas, US Virgin Islands.

## ﻿Erratum

Supplementary Plate S17: The label embedded in the image that reads “*Rypticuscarpenteri*” is incorrect and should read “*Rypticussubbifrenatus*”. The distributions of large dark spots on the interorbital areas and the colors of the fins of those two species differ (Baldwin and Weigt, 2012) and the color pattern of the fish in Plate S17 is that of *R.subbifrenatus*. As that image represents the sole-source voucher of the occurrence of *R.carpenteri* it is removed from the St. John-Thomas inventory.

## ﻿Addition

Fig. 1 here is an image of a Longhorn Blenny, *Hypsoblenniusexstochilus* Bohlke, 1959, taken at Botany Bay (18.3585, -65.0335) at St. Thomas. That species is easily recognized by its diagnostic pair of orbital cirri, each of which consists of a very large, branched stalk, and the color pattern of its head. This image represents the sole-source record for the addition of this species to the St. John-Thomas faunal inventory.

The combination of the removal of *R.carpenteri* from and addition of *H.exstochilus* to the St. John-Thomas inventory had very little effect on data in Tables 3-6: In Table 3 the only change is the addition of one to the number of uncommon shallow species. The only changes in Table 5 are small increases (< 0.5%) in the percentages of Core Coral Reef Fishes. There are no changes to Tables 4, 6.
