# Supplementary figures and images for: ﻿An updated, illustrated inventory of the marine fishes of the US Virgin Islands
Source: Zookeys. 2022 Jun 1;1103:79–122. doi: 10.3897/zookeys.1103.83795 (PMC9848822; doi:10.3897/zookeys.1103.83795)

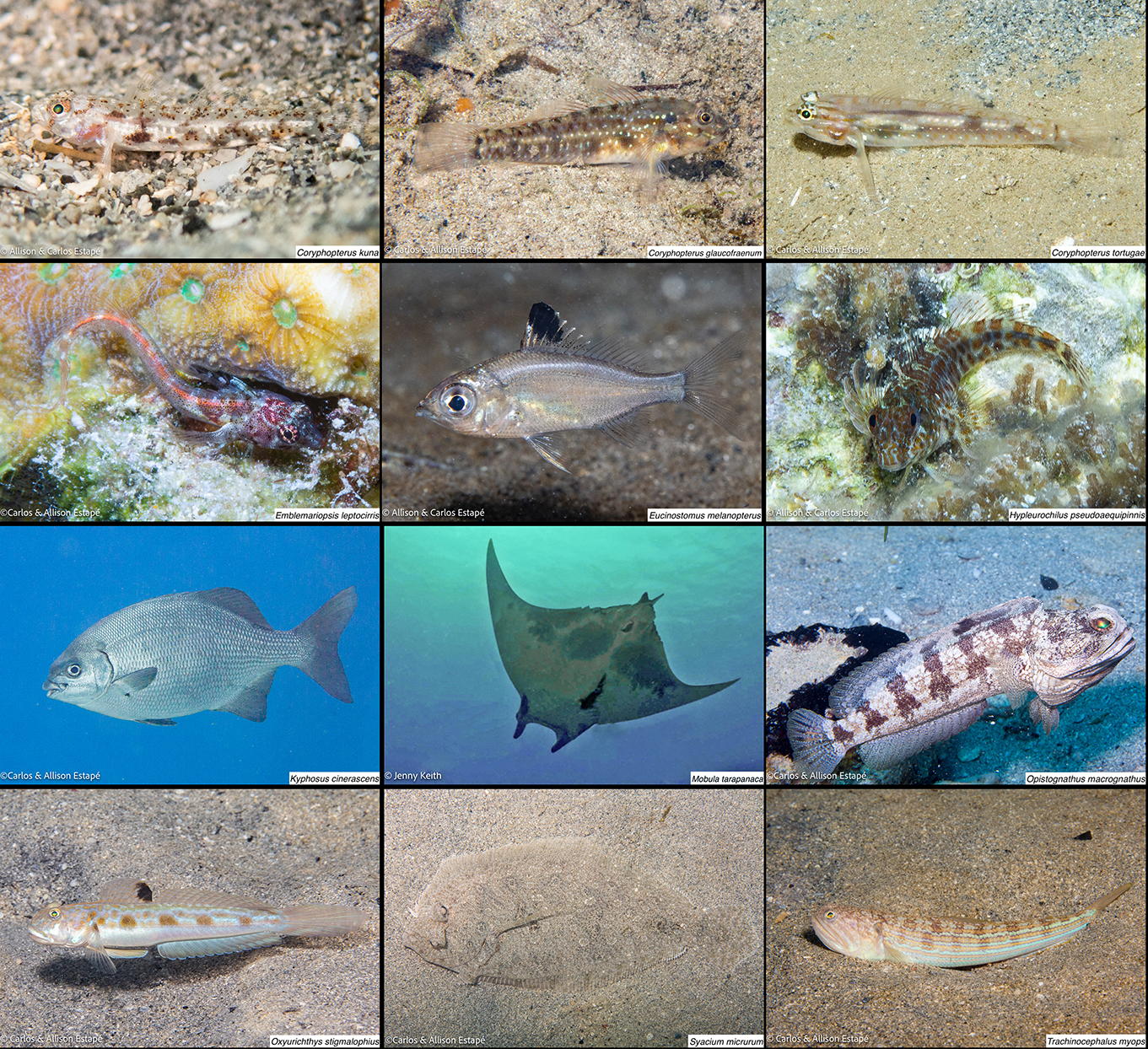

Supplement: Supplementary material 1 — Plates S1–S18 [file zookeys-1103-079-s001.zip › 83795_2L-1-LE_Suppl-material-01/83795_0R-1-A_Plate S1.jpg]

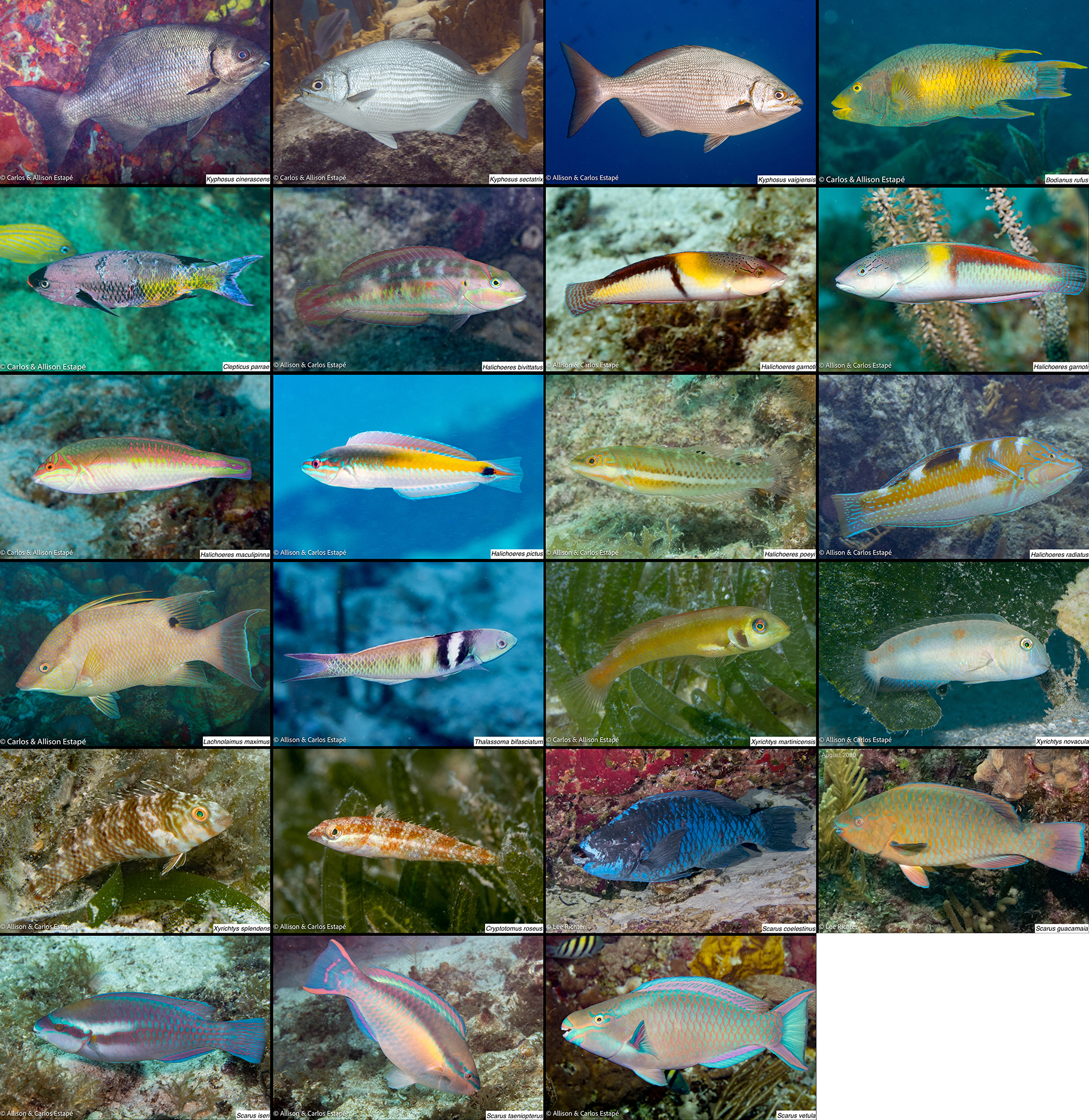

Supplement: Supplementary material 1 — Plates S1–S18 [file zookeys-1103-079-s001.zip › 83795_2L-1-LE_Suppl-material-01/83795_0R-1-A_Plate S10.jpg]

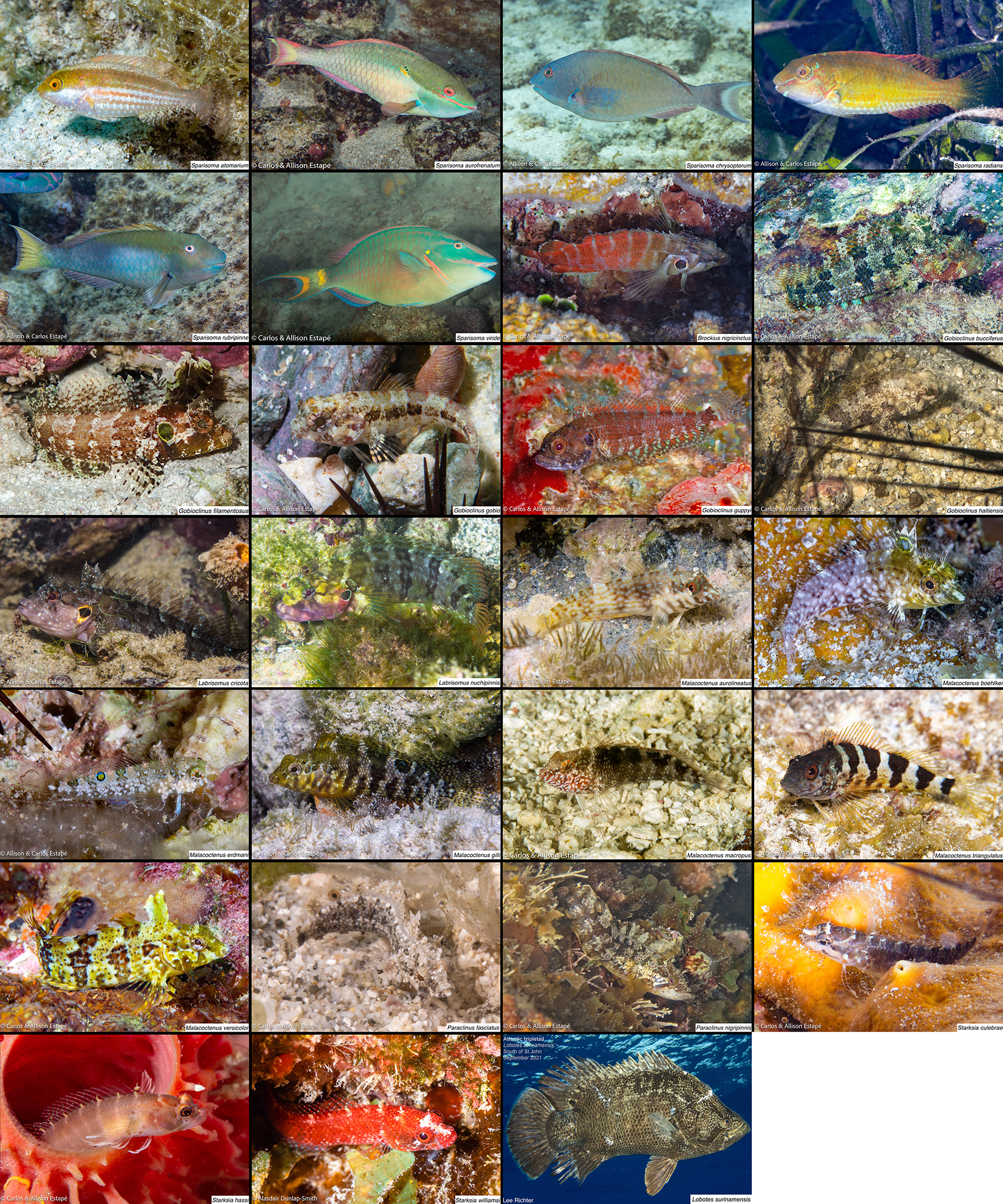

Supplement: Supplementary material 1 — Plates S1–S18 [file zookeys-1103-079-s001.zip › 83795_2L-1-LE_Suppl-material-01/83795_0R-1-A_Plate S11.jpg]

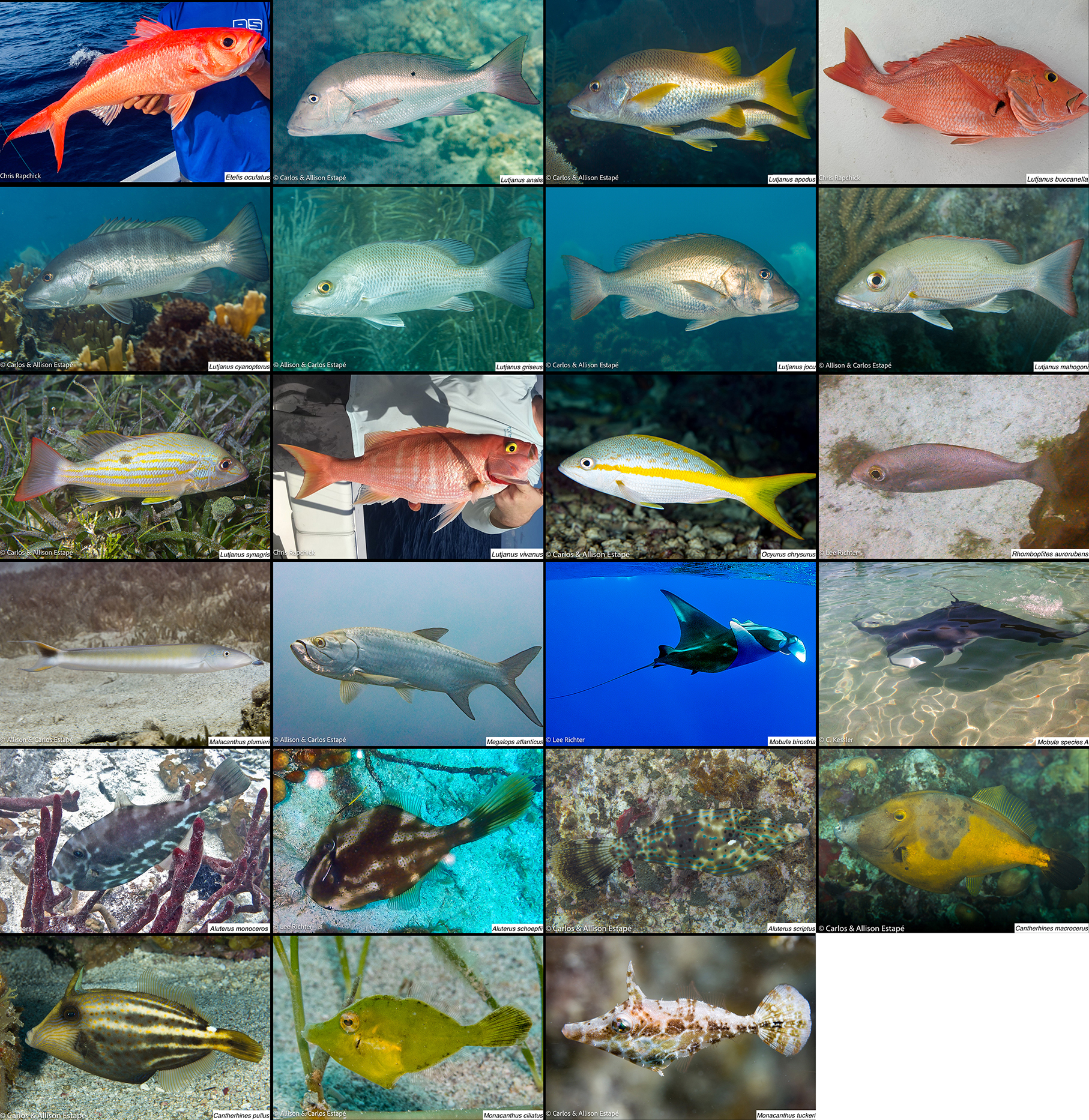

Supplement: Supplementary material 1 — Plates S1–S18 [file zookeys-1103-079-s001.zip › 83795_2L-1-LE_Suppl-material-01/83795_0R-1-A_Plate S12.jpg]

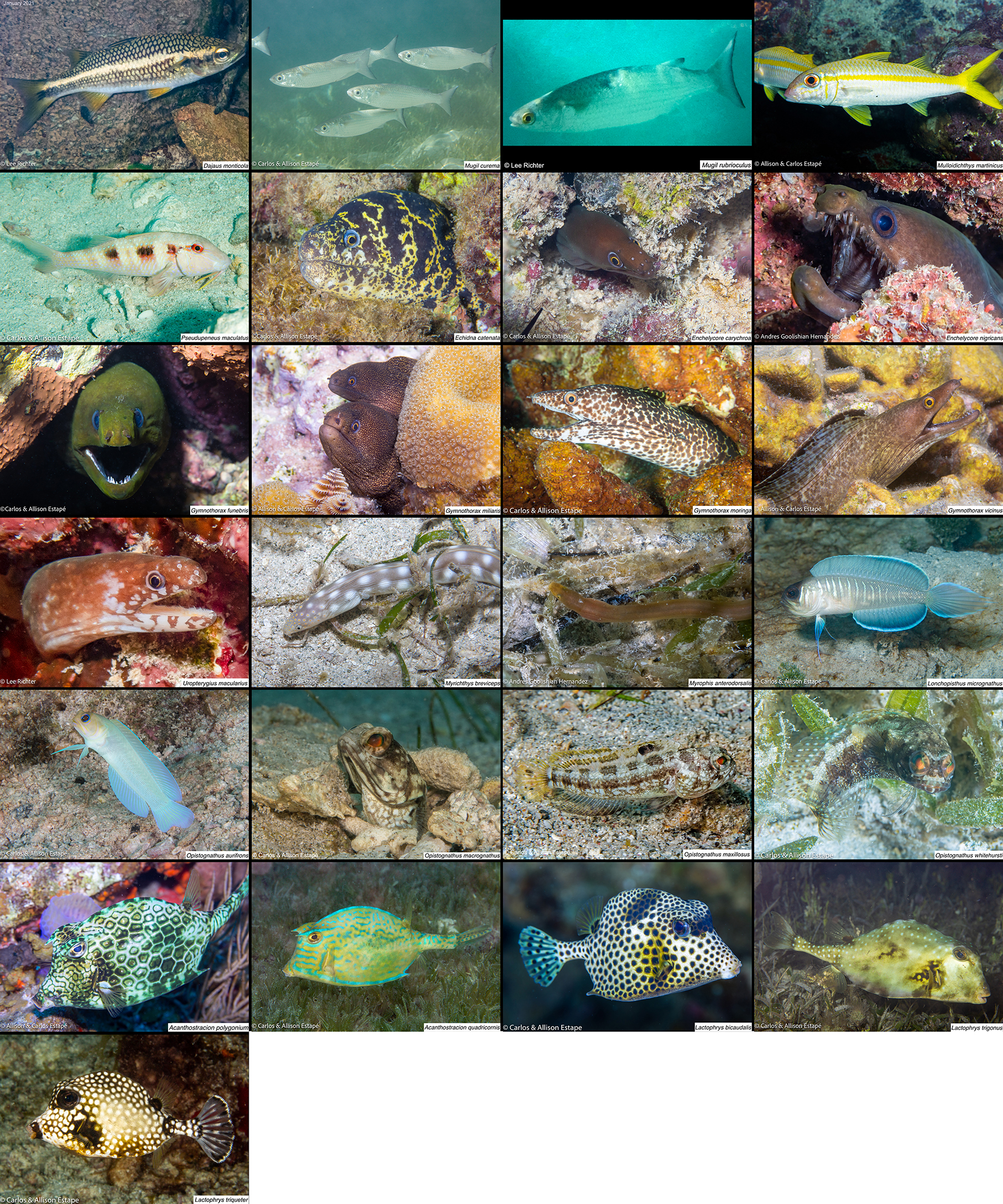

Supplement: Supplementary material 1 — Plates S1–S18 [file zookeys-1103-079-s001.zip › 83795_2L-1-LE_Suppl-material-01/83795_0R-1-A_Plate S13.jpg]

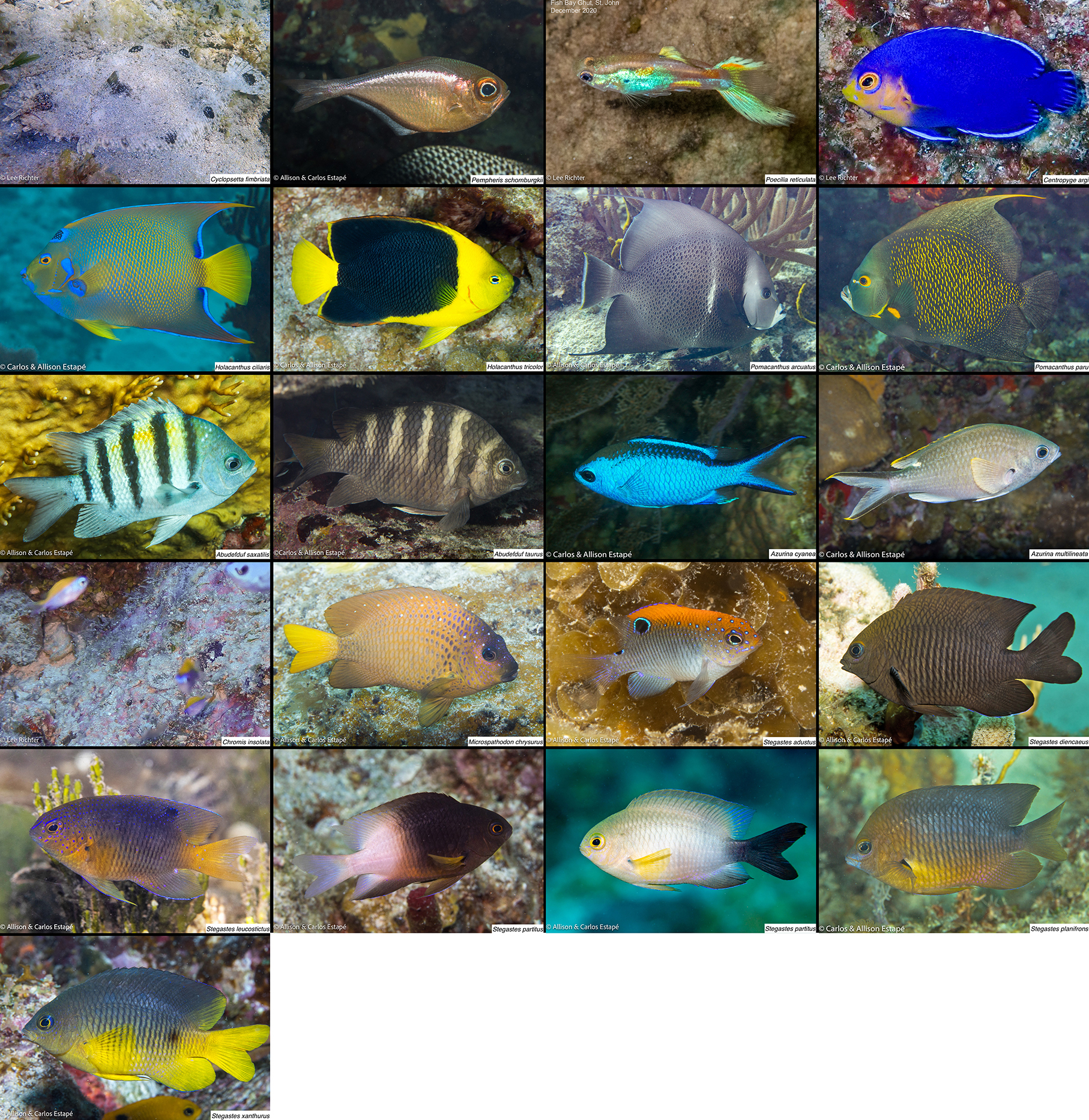

Supplement: Supplementary material 1 — Plates S1–S18 [file zookeys-1103-079-s001.zip › 83795_2L-1-LE_Suppl-material-01/83795_0R-1-A_Plate S14.jpg]

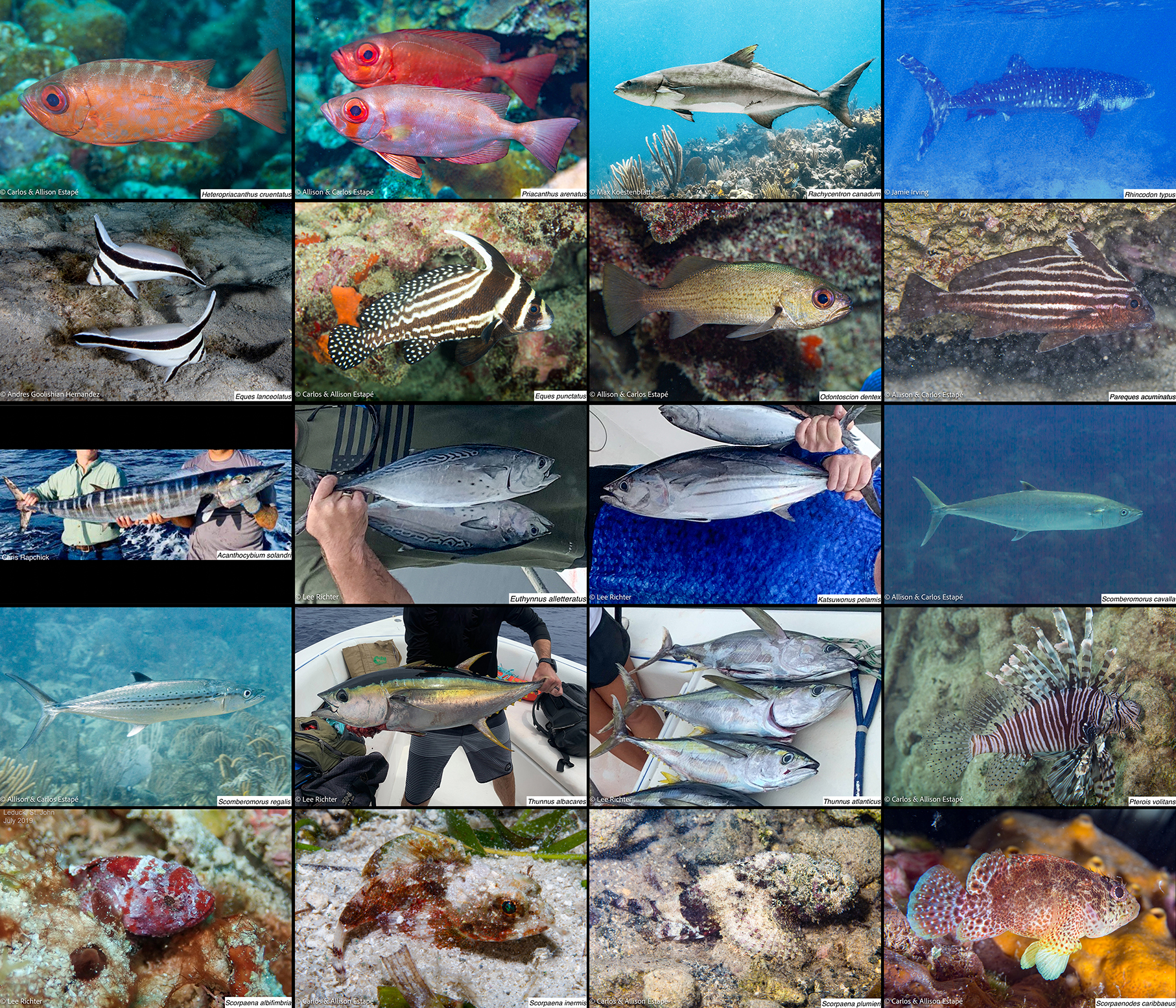

Supplement: Supplementary material 1 — Plates S1–S18 [file zookeys-1103-079-s001.zip › 83795_2L-1-LE_Suppl-material-01/83795_0R-1-A_Plate S15.jpg]

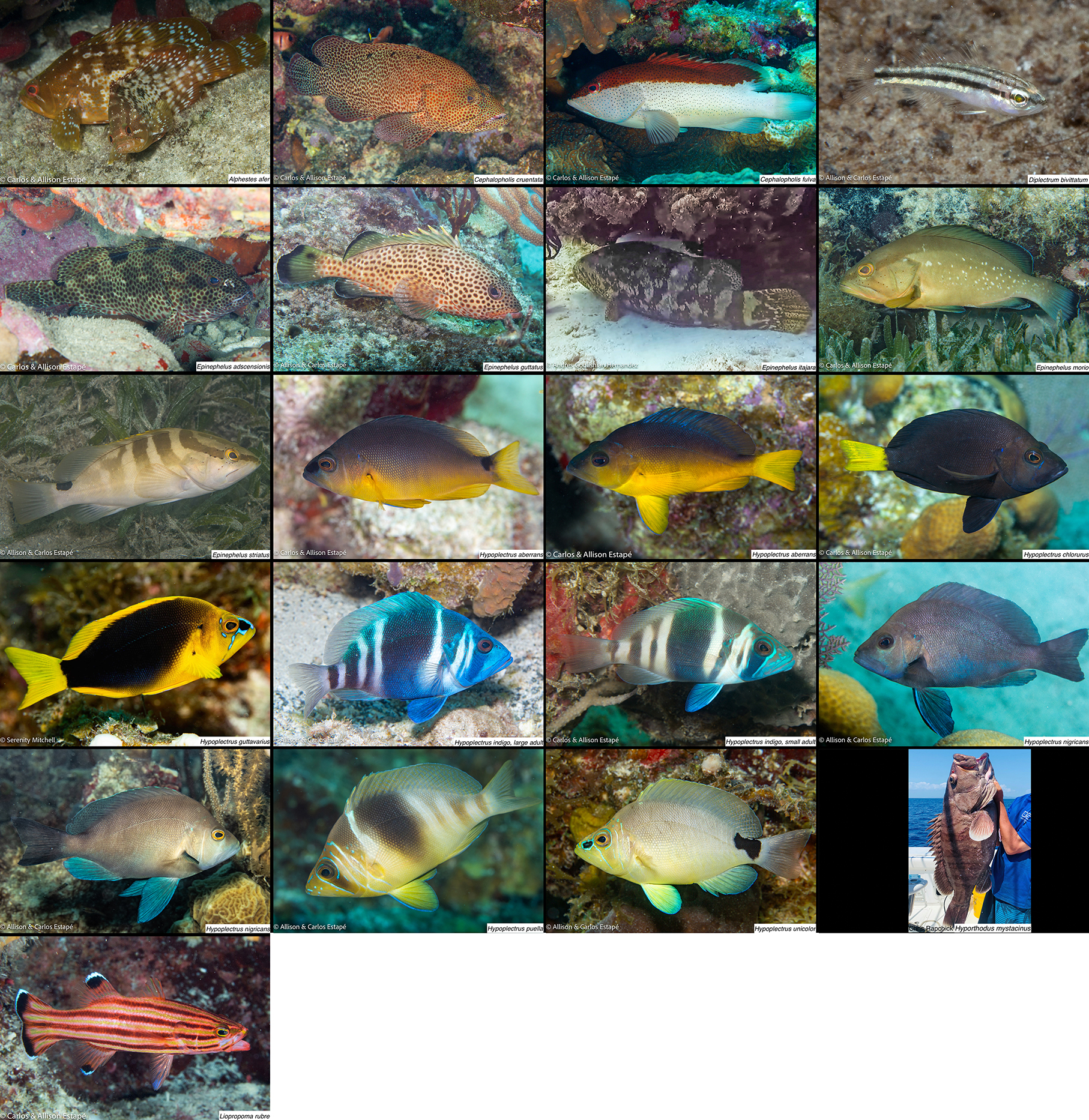

Supplement: Supplementary material 1 — Plates S1–S18 [file zookeys-1103-079-s001.zip › 83795_2L-1-LE_Suppl-material-01/83795_0R-1-A_Plate S16.jpg]

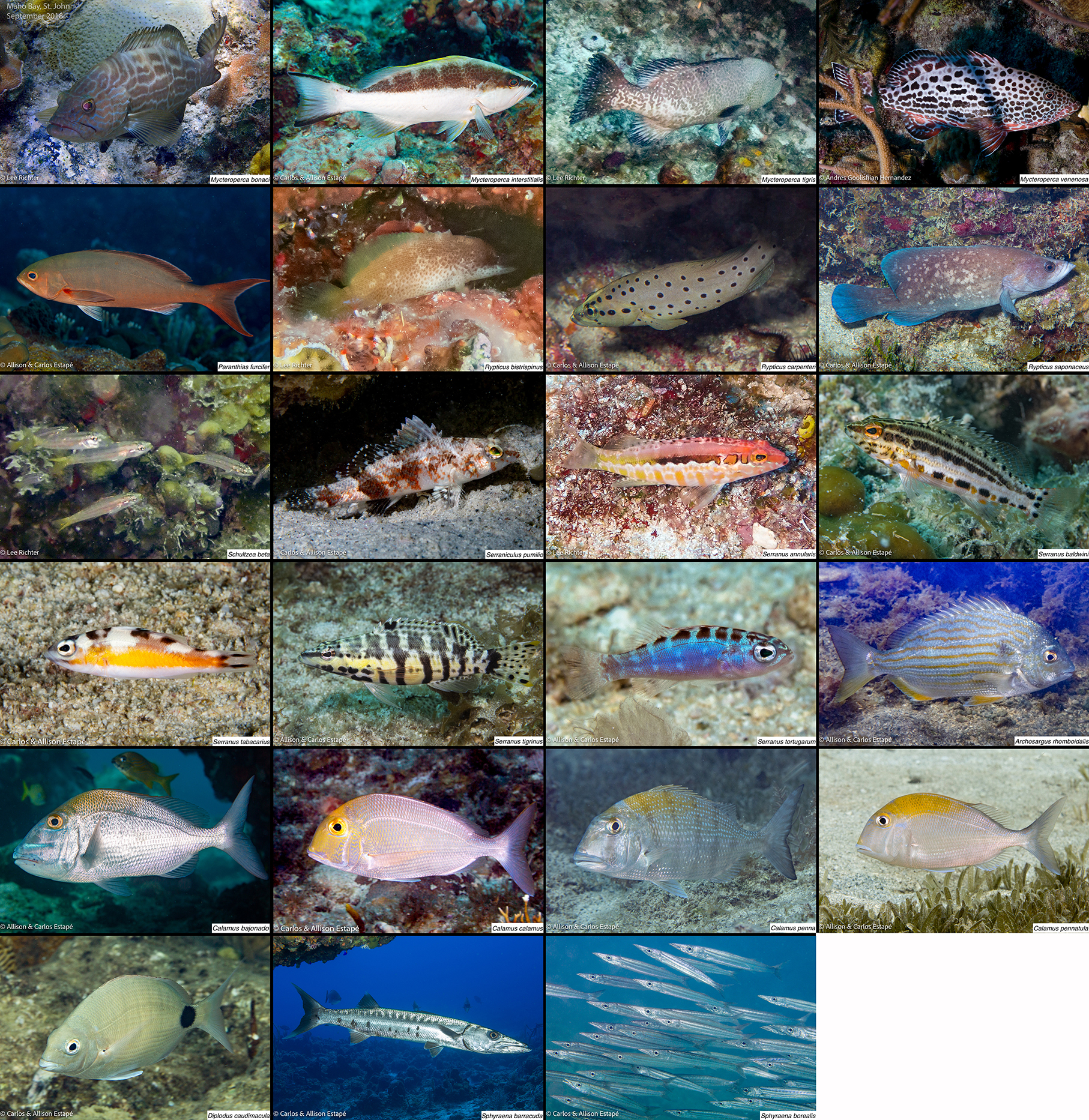

Supplement: Supplementary material 1 — Plates S1–S18 [file zookeys-1103-079-s001.zip › 83795_2L-1-LE_Suppl-material-01/83795_0R-1-A_Plate S17.jpg]

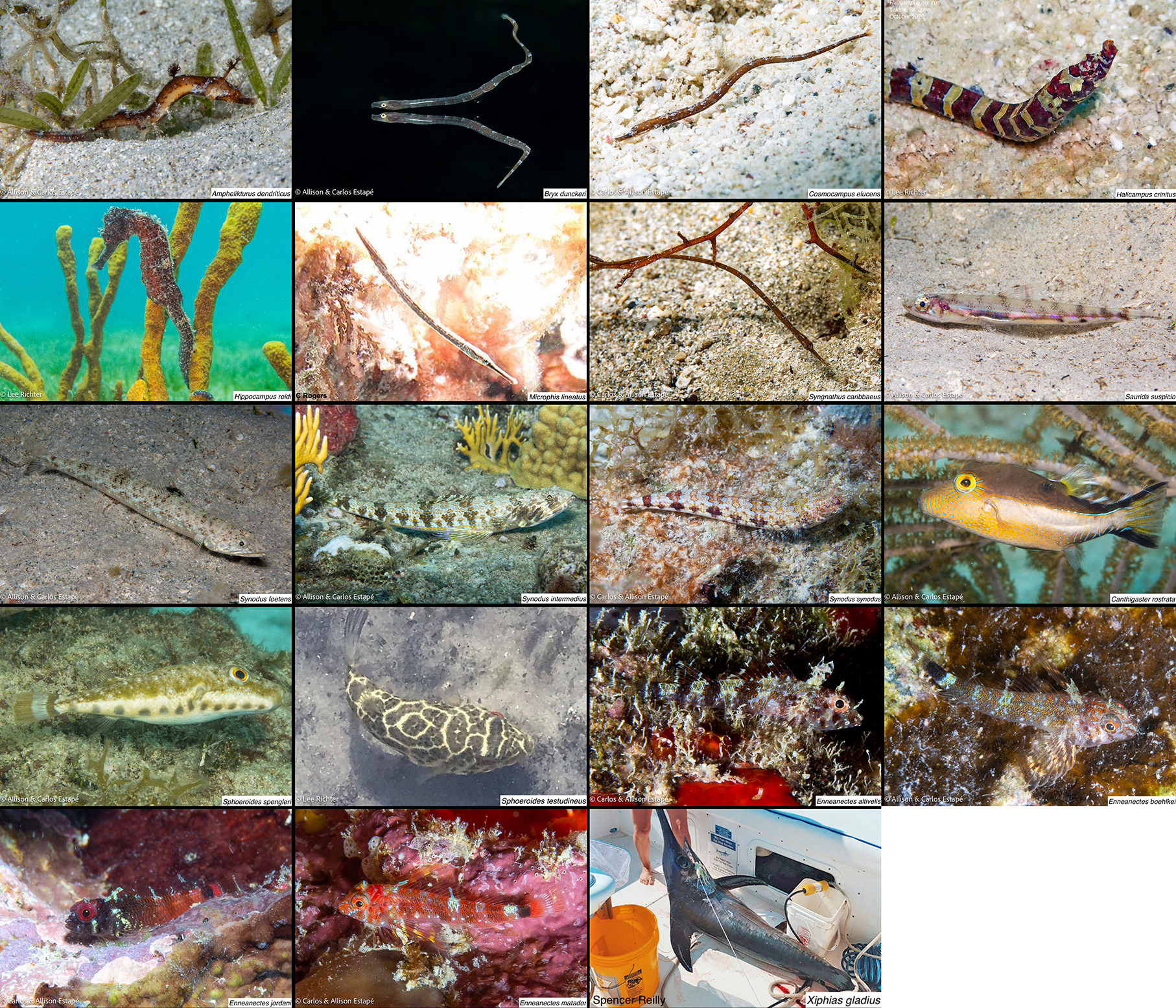

Supplement: Supplementary material 1 — Plates S1–S18 [file zookeys-1103-079-s001.zip › 83795_2L-1-LE_Suppl-material-01/83795_0R-1-A_Plate S18.jpg]

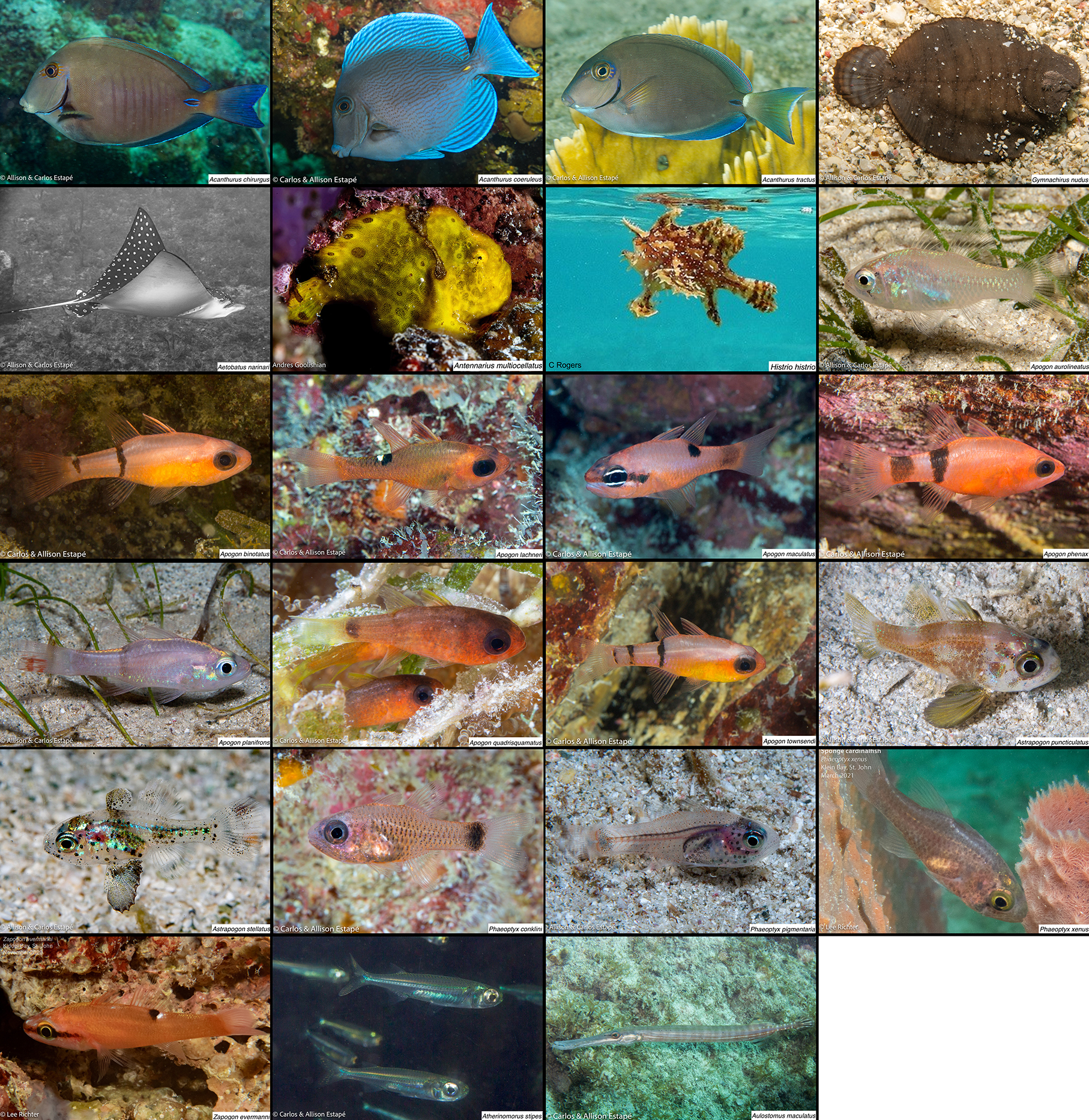

Supplement: Supplementary material 1 — Plates S1–S18 [file zookeys-1103-079-s001.zip › 83795_2L-1-LE_Suppl-material-01/83795_0R-1-A_Plate S2.jpg]

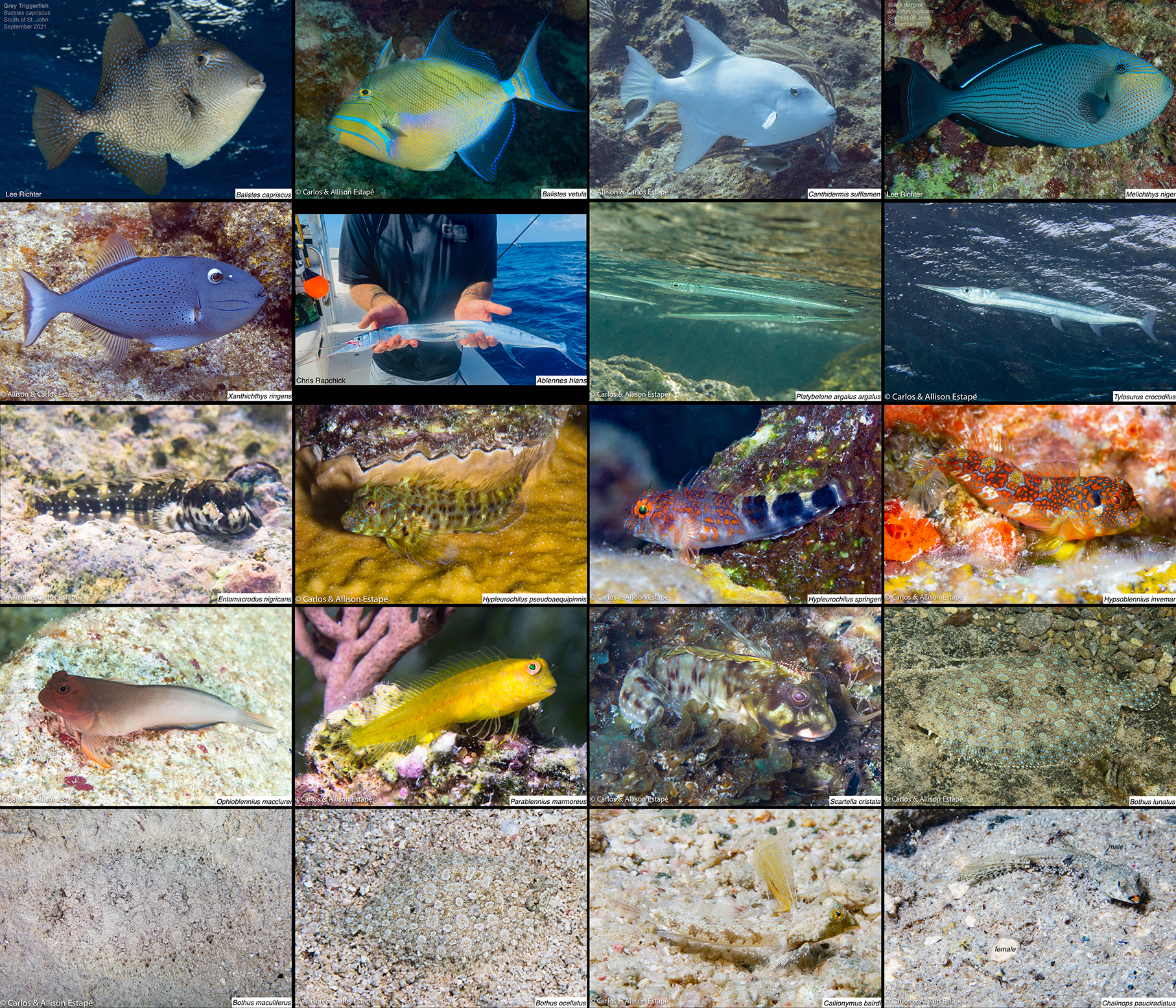

Supplement: Supplementary material 1 — Plates S1–S18 [file zookeys-1103-079-s001.zip › 83795_2L-1-LE_Suppl-material-01/83795_0R-1-A_Plate S3.jpg]

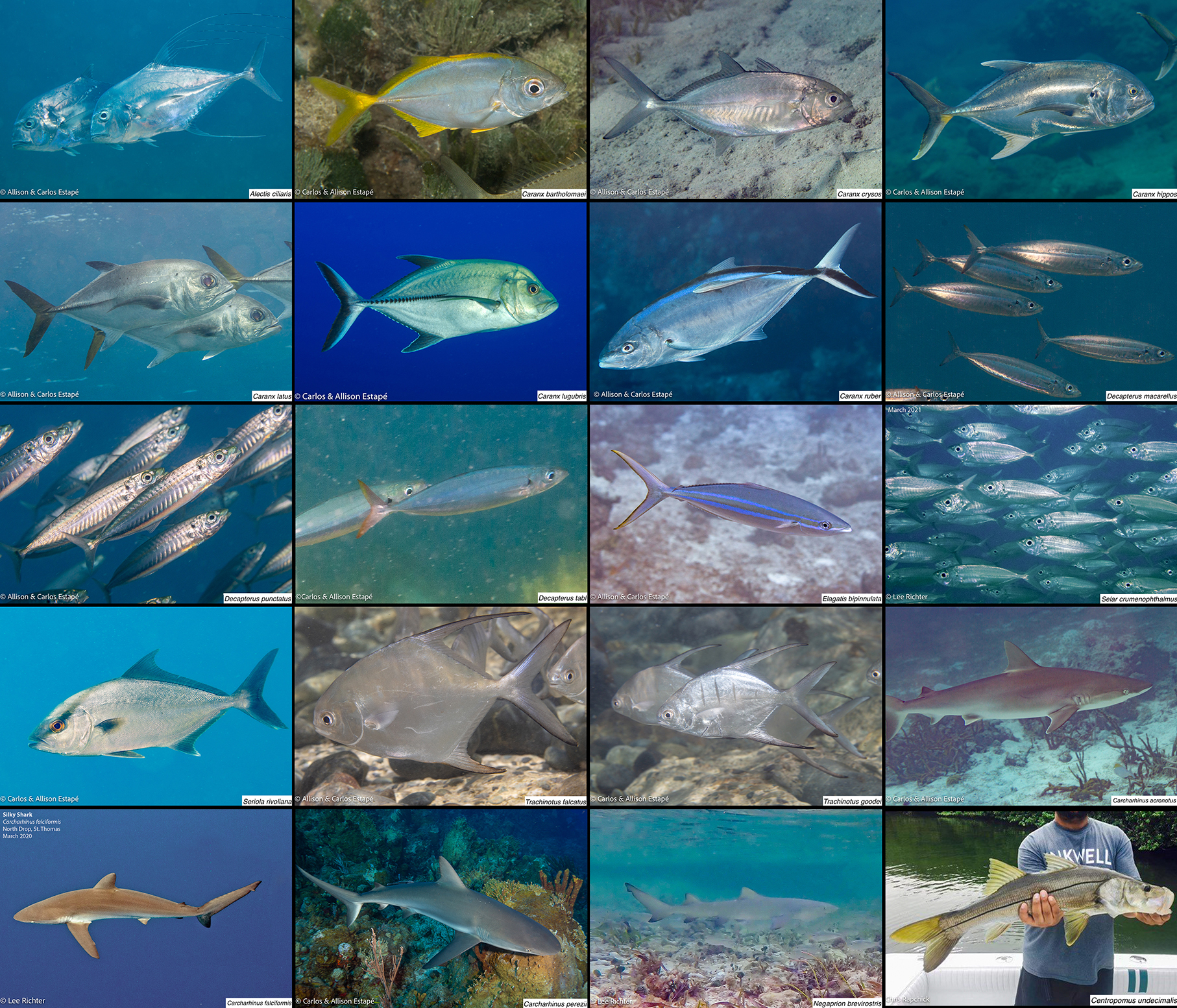

Supplement: Supplementary material 1 — Plates S1–S18 [file zookeys-1103-079-s001.zip › 83795_2L-1-LE_Suppl-material-01/83795_0R-1-A_Plate S4.jpg]

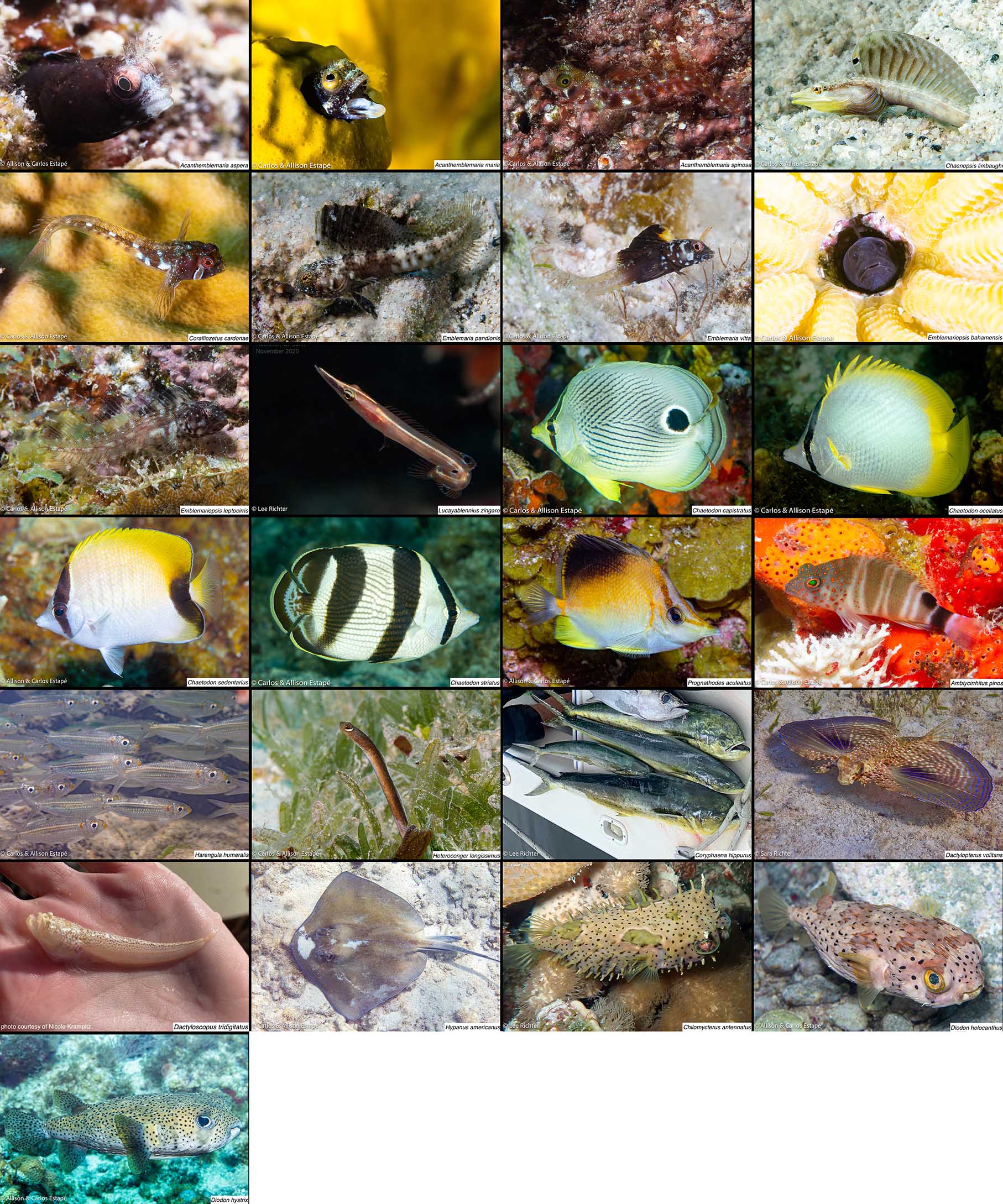

Supplement: Supplementary material 1 — Plates S1–S18 [file zookeys-1103-079-s001.zip › 83795_2L-1-LE_Suppl-material-01/83795_0R-1-A_Plate S5.jpg]

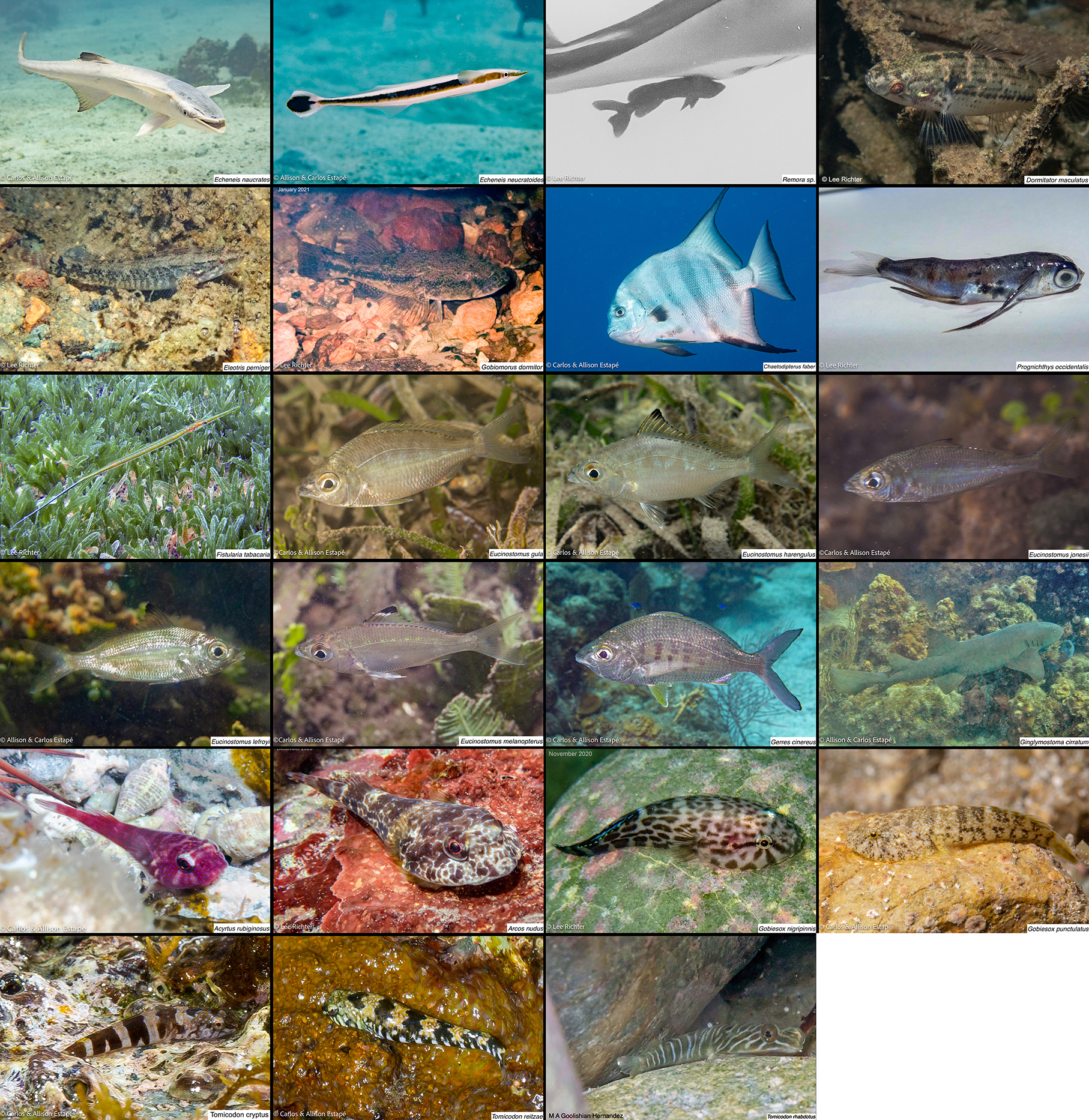

Supplement: Supplementary material 1 — Plates S1–S18 [file zookeys-1103-079-s001.zip › 83795_2L-1-LE_Suppl-material-01/83795_0R-1-A_Plate S6.jpg]

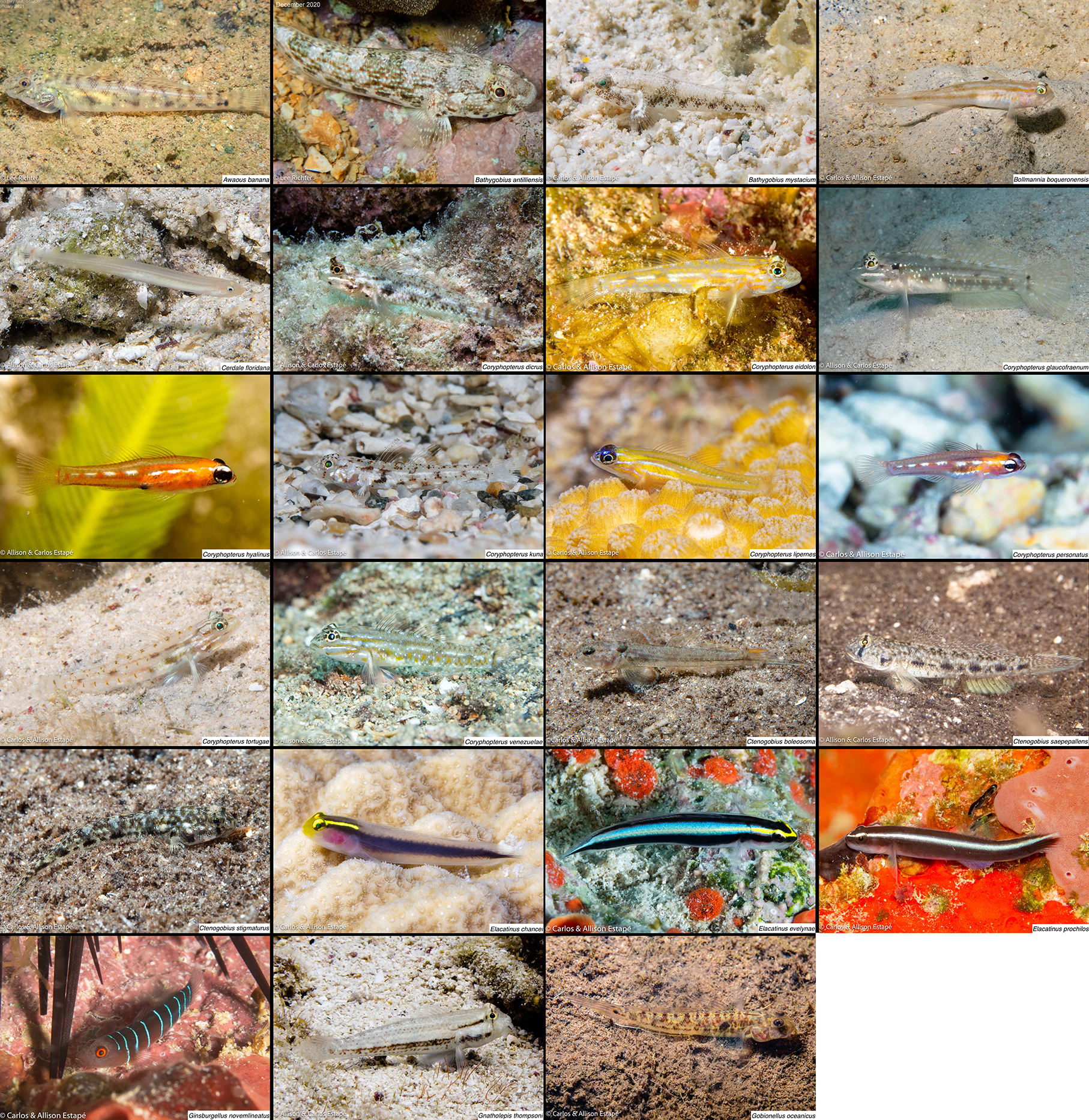

Supplement: Supplementary material 1 — Plates S1–S18 [file zookeys-1103-079-s001.zip › 83795_2L-1-LE_Suppl-material-01/83795_0R-1-A_Plate S7.jpg]

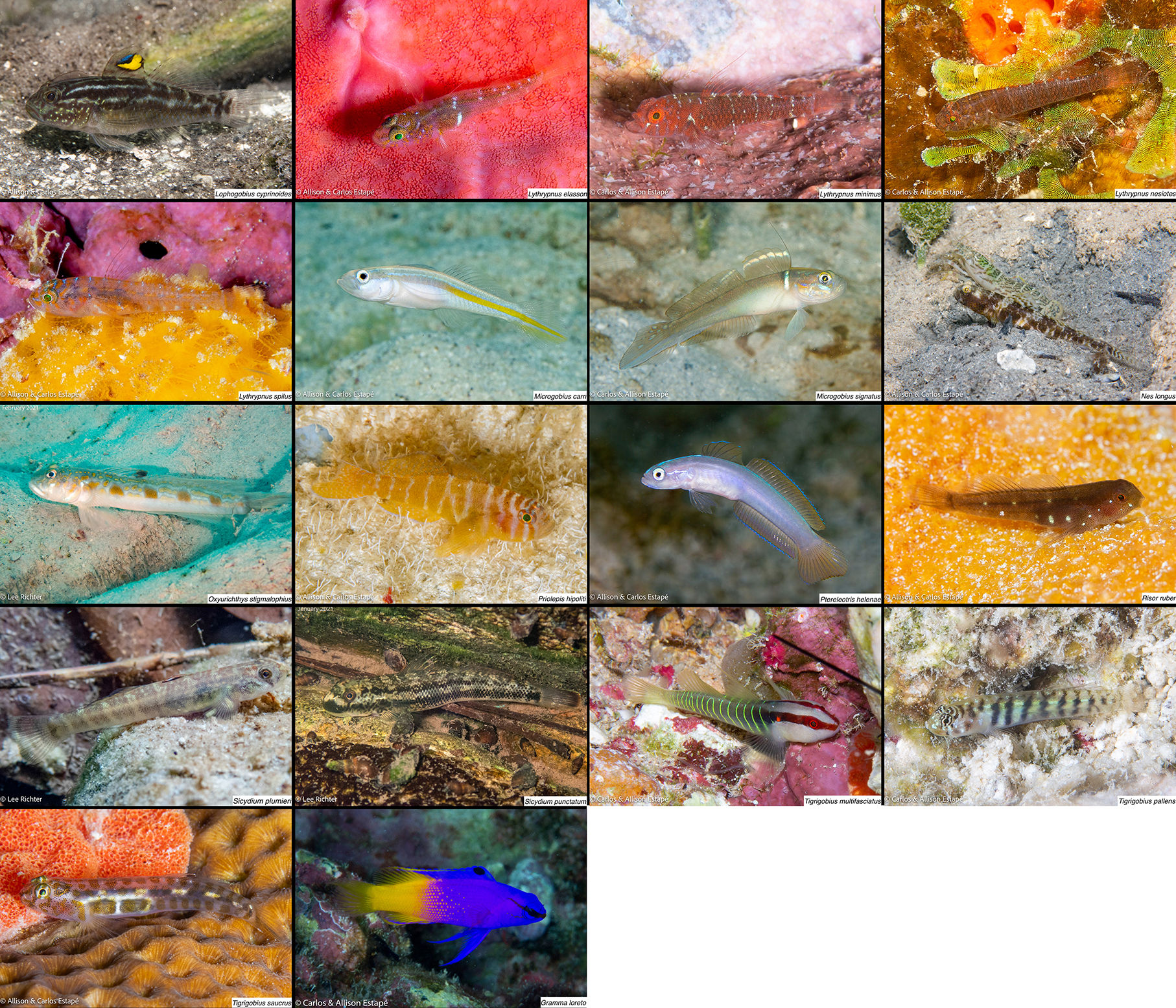

Supplement: Supplementary material 1 — Plates S1–S18 [file zookeys-1103-079-s001.zip › 83795_2L-1-LE_Suppl-material-01/83795_0R-1-A_Plate S8.jpg]

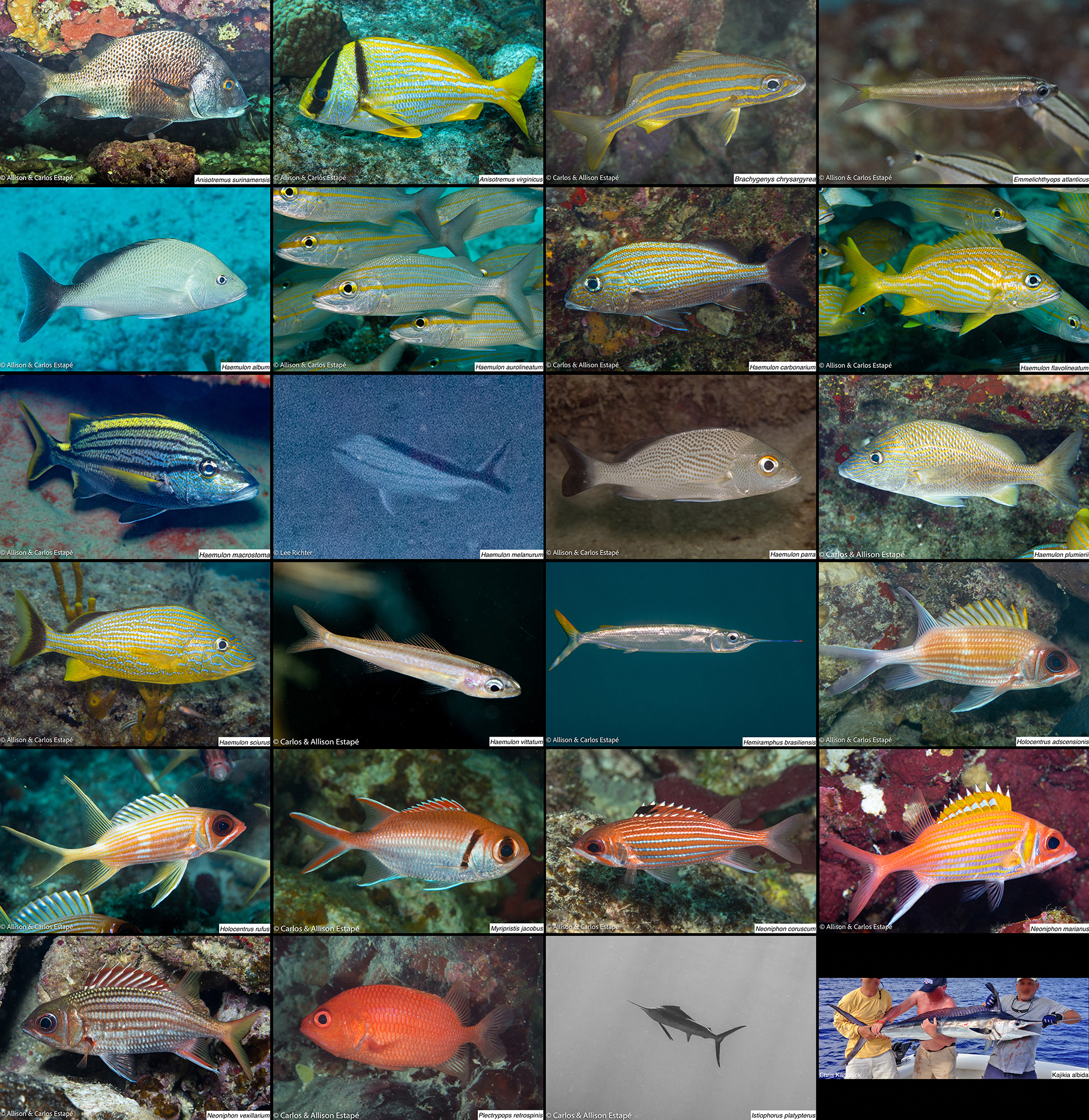

Supplement: Supplementary material 1 — Plates S1–S18 [file zookeys-1103-079-s001.zip › 83795_2L-1-LE_Suppl-material-01/83795_0R-1-A_Plate S9.jpg]

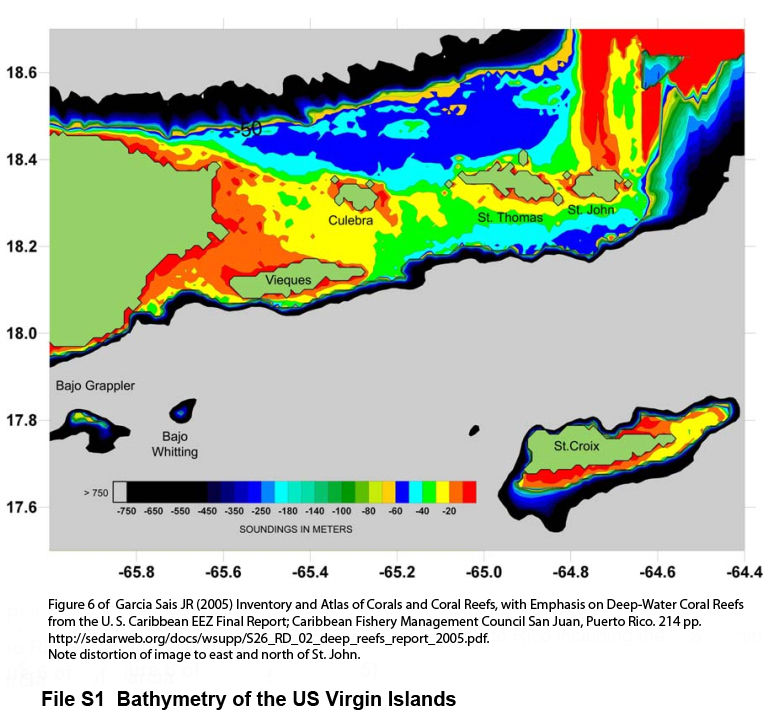

Supplement: Supplementary material 2 — File S1 [file zookeys-1103-079-s002.jpg]
